# On a Variational Definition for the Jensen-Shannon Symmetrization of Distances Based on the Information Radius

**DOI:** 10.3390/e23040464

**Published:** 2021-04-14

**Authors:** Frank Nielsen

**Affiliations:** Sony Computer Science Laboratories, Tokyo 141-0022, Japan; Frank.Nielsen@acm.org

**Keywords:** Jensen-Shannon divergence, diversity index, Rényi entropy, information radius, information projection, exponential family, Bregman divergence, Fenchel–Young divergence, Bregman information, q-exponential family, q-divergence, Bhattacharyya distance, centroid, clustering

## Abstract

We generalize the Jensen-Shannon divergence and the Jensen-Shannon diversity index by considering a variational definition with respect to a generic mean, thereby extending the notion of Sibson’s information radius. The variational definition applies to any arbitrary distance and yields a new way to define a Jensen-Shannon symmetrization of distances. When the variational optimization is further constrained to belong to prescribed families of probability measures, we get relative Jensen-Shannon divergences and their equivalent Jensen-Shannon symmetrizations of distances that generalize the concept of information projections. Finally, we touch upon applications of these variational Jensen-Shannon divergences and diversity indices to clustering and quantization tasks of probability measures, including statistical mixtures.

## 1. Introduction: Background and Motivations

The goal of the author is to methodologically contribute to an extension of the Sibson’s information radius [1] and also concentrate on analysis of the specified families of distributions called exponential families [2].

Let (X,F) denote a measurable space [3] with sample space X and σ-algebra F on the set X. The Jensen-Shannon divergence [4] (JSD) between two probability measures *P* and *Q* (or probability distributions) on (X,F) is defined by:

(1) $$D_{\mathrm{JS}}\left[P,Q\right]:=\frac{1}{2}\left(D_{\mathrm{KL}}\left[P:\frac{P+Q}{2}\right]+D_{\mathrm{KL}}\left[Q:\frac{P+Q}{2}\right]\right),$$

where $D_{\mathrm{KL}}$ denotes the Kullback–Leibler divergence [5,6] (KLD):

(2) $$D_{\mathrm{KL}}\left[P:Q\right]:=\left\{\begin{array}{cc} \int_{X}\log\left(\frac{dP(x)}{dQ(x)}\right)dP, & P\ll Q\\ +\infty , & P\ll Q \end{array}\right.$$

where P≪Q means that *P* is absolutely continuous with respect to *Q* [3], and $\frac{dP}{dQ}$ is the Radon–Nikodym derivative of *P* with respect to *Q*. Equation (2) can be rewritten using the chain rule as:

(3) $$D_{\mathrm{KL}}\left[P:Q\right]:=\left\{\begin{array}{cc} \int_{X}\frac{dP(x)}{dQ(x)}\log\left(\frac{dP(x)}{dQ(x)}\right)dQ, & P\ll Q\\ +\infty , & P\ll Q \end{array}\right.$$

Consider a measure μ for which both the Radon–Nikodym derivatives $p:=\frac{dP}{d\mu }$ and $q:=\frac{dP}{d\mu }$ exist (e.g., $\mu =\frac{P+Q}{2}$). Subsequently the Kullback–Leibler divergence can be rewritten as (see Equation (2.5) page 5 of [5] and page 251 of the Cover & Thomas’ textbook [6]):

(4) $$D_{\mathrm{KL}}\left[p:q\right]:=\int_{X}p\left(x\right)\log\left(\frac{p(x)}{q(x)}\right)d\mu \left(x\right).$$

Denote by D=D(X) the set of all densities with full support X (Radon–Nikodym derivatives of probability measures with respect to μ):

$$D\left(X\right):=\left\{p\;:\;X\to R\;:\;p\left(x\right)>0\;\mu -almost\;everywhere,\int_{X}p\left(x\right)d\mu \left(x\right)=1\right\}.$$

Subsequently, the Jensen-Shannon divergence [4] between two densities *p* and *q* of D is defined by:

(5) $$D_{\mathrm{JS}}\left[p,q\right]:=\frac{1}{2}\left(D_{\mathrm{KL}}\left[p:\frac{p+q}{2}\right]+D_{\mathrm{KL}}\left[q:\frac{p+q}{2}\right]\right).$$

Often, one considers the Lebesgue measure [3] $\mu =\mu_{L}$ on $\left(R^{d},B\left(R^{d}\right)\right)$, where $B(R^{d})$ is the Borel σ-algebra, or the counting measure [3]$\mu =\mu_{\#}$ on $\left(X,2^{X}\right)$ where X is a countable set, for defining the measure space (X,F,μ).

The JSD belongs to the class of *f*-divergences [7,8,9] which are known as the invariant decomposable divergences of information geometry (see [10], pp. 52–57). Although the KLD is asymmetric (i.e., $D_{\mathrm{KL}}\left[p:q\right]\neq D_{\mathrm{KL}}\left[q:p\right]$), the JSD is symmetric (i.e., $D_{\mathrm{JS}}\left[p,q\right]=D_{\mathrm{JS}}\left[q,p\right]$). The notation ‘:’ is used as a parameter separator to indicate that the parameters are not permutation invariant, and that the order of parameters is important.

In this work, a distance $D(O_{1}:O_{2})$ is a measure of dissimilarity between two objects $O_{1}$ and $O_{2}$, which do not need to be symmetric or satisfy the triangle inequality of metric distances. A distance only satisfies the identity of indiscernibles: $D(O_{1}:O_{2})=0$ if and only if $O_{1}=O_{2}$. When the objects $O_{1}$ and $O_{2}$ are probability densities with respect to μ, we call this distance a statistical distance, use the brackets to enclose the arguments of the statistical distance (i.e., $D[O_{1}:O_{2}]$), and we have $D[O_{1}:O_{2}]=0$ if and only if $O_{1}\left(x\right)=O_{2}\left(x\right)$μ-almost everywhere.

The 2-point JSD of Equation (4) can be extended to a weighted set of *n* densities $P:=\{\left(w_{1},p_{1}\right),\ldots ,\left(w_{n},p_{n}\right)\}$ (with positive $w_{i}$’s normalized to sum up to unity, i.e., $\sum_{i=1}^{n}w_{i}=1$) thus providing a diversity index, i.e., a *n*-point JSD for P:

(6) $$D_{\mathrm{JS}}\left(P\right):=\sum_{i=1}^{n}w_{i}D_{\mathrm{KL}}\left[p_{i}:\bar{p}\right],$$

where $\bar{p}:=\sum_{i=1}^{n}w_{i}p_{i}$ denotes the statistical mixture [11] of the densities of P. We have $D_{\mathrm{JS}}\left[p:q\right]=D_{\mathrm{JS}}\left(\left\{\left(\frac{1}{2},p\right),\left(\frac{1}{2},q\right)\right\}\right)$. We call $D_{\mathrm{JS}}\left(P\right)$ the Jensen-Shannon diversity index.

The KLD is also called the relative entropy since it can be expressed as the difference between the cross entropy h[p:q] and the entropy h[p]:

(7) $$\begin{array}{ccc} D_{\mathrm{KL}}\left[p:q\right] & := & \int_{X}p\left(x\right)\log\left(\frac{p(x)}{q(x)}\right)d\mu \left(x\right) \end{array}$$

(8) $$\begin{array}{ccc} & = & \int_{X}p\left(x\right)\log p(x)d\mu \left(x\right)-\int_{X}p\left(x\right)\log q(x)d\mu \left(x\right), \end{array}$$

(9) $$\begin{array}{ccc} & = & h[p:q]-h[p], \end{array}$$

with the cross-entropy and entropy defined, respectively, by

(10) $$\begin{array}{ccc} h[p:q] & := & -\int_{X}p\left(x\right)\log q\left(x\right)d\mu \left(x\right), \end{array}$$

(11) $$\begin{array}{ccc} h[p] & := & -\int_{X}p\left(x\right)\log p\left(x\right)d\mu \left(x\right). \end{array}$$

Because h[p]=h[p:p], we may say that the entropy is the self-cross-entropy.

When μ is the Lebesgue measure, the Shannon entropy is also called the differential entropy [6]. Although the discrete entropy $H\left[p\right]=-\sum_{i}p_{i}\log p_{i}$ (i.e., entropy with respect to the counting measure) is always positive and bounded by log|X|, the differential entropy may be negative (e.g., entropy of a Gaussian distribution with small variance).

The Jensen-Shannon divergence of Equation (6) can be rewritten as:

(12) $$D_{\mathrm{JS}}\left[p,q\right]=h\left[\bar{p}\right]-\sum_{i=1}^{n}w_{i}h\left[p_{i}\right]:=J_{-h}\left[p,q\right].$$

The JSD representation of Equation (12) is a Jensen divergence [12] for the strictly convex negentropy F(p)=−h[p], since the entropy function h[.] is strictly concave. Therefore, it is appropriate to call this divergence the Jensen-Shannon divergence.

Because $\frac{p_{i}\left(x\right)}{\bar{p}\left(x\right)}\leq \frac{p_{i}\left(x\right)}{w_{i}p_{i}\left(x\right)}=\frac{1}{w_{i}}$, it can be shown that the Jensen-Shannon diversity index is upper bounded by $H\left(w\right):=-\sum_{i=1}^{n}w_{i}\log w_{i}$, the discrete Shannon entropy. Thus, the Jensen-Shannon diversity index is bounded by logn, and the 2-point JSD is bounded by log2, although the KLD is unbounded and it may even be equal to +∞ when the definite integral diverges (e.g., KLD between the standard Cauchy distribution and the standard Gaussian distribution). Another nice property of the JSD is that its square root yields a metric distance [13,14]. This property further holds for the quantum JSD [15]. The JSD has gained interest in machine learning. See, for example, the Generative Adversarial Networks [16] (GANs) in deep learning [17], where it was proven that minimizing the GAN objective function by adversarial training is equivalent to minimizing a JSD.

To delineate the different roles that are played by the factor $\frac{1}{2}$ in the ordinary Jensen-Shannon divergence (i.e., in weighting the two KLDs and in weighting the two densities), let us introduce two scalars α,β∈(0,1), and define a generic (α,β)-skewed Jensen-Shannon divergence, as follows:

(13) $$\begin{array}{ccc} D_{\mathrm{JS},\alpha ,\beta }\left[p:q\right] & := & \left(1-\beta \right)D_{\mathrm{KL}}\left[p:m_{\alpha }\right]+\beta D_{\mathrm{KL}}\left[q:m_{\alpha }\right], \end{array}$$

(14) $$\begin{array}{ccc} & = & \left(1-\beta \right)h\left[p:m_{\alpha }\right]+\beta h\left[q:m_{\alpha }\right]-\left(1-\beta \right)h\left[p\right]-\beta h\left[q\right], \end{array}$$

(15) $$\begin{array}{ccc} & = & h\left[m_{\beta }:m_{\alpha }\right]-\left((1-\beta )h[p]+\beta h[q]\right), \end{array}$$

where $m_{\alpha }:=\left(1-\alpha \right)p+\alpha q$ and $m_{\beta }:=\left(1-\beta \right)p+\beta q$. This identity holds, because $D_{\mathrm{JS},\alpha ,\beta }$ is bounded by $\left(1-\beta \right)\log\frac{1}{1-\alpha }+\beta \log\frac{1}{\alpha }$, see [18]. Thus, when β=α, we have $D_{\mathrm{JS},\alpha }\left[p,q\right]=D_{\mathrm{JS},\alpha ,\alpha }\left[p,q\right]=h\left[m_{\alpha }\right]-\left(\left(1-\alpha \right)h\left[p\right]+\alpha h\left[q\right]\right)$, since the self-cross entropy corresponds to the entropy: $h\left[m_{\alpha }:m_{\alpha }\right]=h\left[m_{\alpha }\right]$.

A *f*-divergence [9,19,20] is defined for a convex generator *f*, which is strictly convex at 1 (to satisfy the identity of the indiscernibles) and that satisfies f(1)=0, by

(16) $$I_{f}\left[p:q\right]:=\int p\left(x\right)f\left(\frac{q(x)}{p(x)}\right)d\mu \left(x\right)\geq f\left(1\right)=0,$$

where the right-hand-side follows from Jensen’s inequality [20]. For example, the total variation distance $D_{\mathrm{TV}}\left[p:q\right]=\frac{1}{2}\int_{X}\left|p\left(x\right)-q\left(x\right)\right|d\mu \left(x\right)$ is a *f*-divergence for the generator $f_{\mathrm{TV}}\left(u\right)=\left|u-1\right|$: $D_{\mathrm{TV}}\left[p:q\right]=I_{f_{\mathrm{TV}}}\left[p:q\right]$. The generator $f_{\mathrm{TV}}\left(u\right)$ is convex on R, strictly convex at 1, and it satisfies f(u)=1.

The $D_{\mathrm{JS},\alpha ,\beta }$ divergence is a *f*-divergence

(17) $$D_{\mathrm{JS},\alpha ,\beta }\left[p:q\right]=I_{f_{\mathrm{JS},\alpha ,\beta }}\left[p:q\right],$$

for the generator:

(18) $$f_{\mathrm{JS},\alpha ,\beta }\left(u\right)=-\left(\left(1-\beta \right)\log\left(\alpha u+(1-\alpha )\right)+\beta u\log\left(\frac{1-\alpha }{u}+\alpha \right)\right).$$

We check that the generator $f_{\mathrm{JS},\alpha ,\beta }$ is strictly convex, since, for any a∈(0,1) and b∈(0,1), we have

(19) $$f_{\mathrm{JS},\alpha ,\beta }^{\prime \prime }\left(u\right)=\frac{a^{2}\left(1-b\right)u+{\left(a-1\right)}^{2}b}{a^{2}u^{3}+2a\left(1-a\right)u^{2}+{\left(a-1\right)}^{2}u}>0,$$

when u>0.

The Jensen-Shannon principle of taking the average of the (Kullback–Leibler) divergences between the source parameters to the mid-parameter can be applied to other distances. For example, the Jensen–Bregman divergence is a Jensen-Shannon symmetrization of the Bregman divergence $B_{F}$ [12]:

(20) $$B_{F}^{\mathrm{JS}}\left(\theta_{1}:\theta_{2}\right):=\frac{1}{2}\left(B_{F}\left(\theta_{1}:\frac{\theta_{1}+\theta_{2}}{2}\right)+B_{F}\left(\theta_{2}:\frac{\theta_{1}+\theta_{2}}{2}\right)\right),$$

where the Bregman divergence [21] $B_{F}$ is defined by

(21) $$B_{F}\left(\theta :\theta^{\prime }\right):=F\left(\theta \right)-F\left(\theta^{\prime }\right)-{\left(\theta -\theta^{\prime }\right)}^{⊤}\nabla F\left(\theta^{\prime }\right).$$

The Jensen–Bregman divergence $B_{F}^{\mathrm{JS}}$ can also be written as an equivalent Jensen divergence $J_{F}$:

(22) $$B_{F}^{\mathrm{JS}}\left(\theta_{1}:\theta_{2}\right)=J_{F}\left(\theta_{1}:\theta_{2}\right):=\frac{F\left(\theta_{1}\right)+F\left(\theta_{2}\right)}{2}-F\left(\frac{\theta_{1}+\theta_{2}}{2}\right),$$

where *F* is a strictly convex function ensuring $J_{F}\left(\theta_{1}:\theta_{2}\right)\geq 0$ with equality if $\theta_{1}=\theta_{2}$.

Because of its use in various fields of information sciences [22], various generalizations of the JSD have been proposed: These generalizations are either based on Equation (5) [23] or Equation (12) [18,24,25]. For example, the (arithmetic) mixture $\bar{p}=\sum_{i}w_{i}p_{i}$ in Equation (6) was replaced by an abstract statistical mixture with respect to a generic mean *M* in [23] (e.g., the geometric mixture induced by the geometric mean), and the two KLDS defining the JSD in Equation (5) was further averaged using another abstract mean *N*, thus yielding the following generic *(M,N)-Jensen-Shannon divergence* [23] (abbreviated as (M,N)-JSD):

(23) $$D_{\mathrm{JS}}^{M,N}\left[p:q\right]:=N\left(D_{\mathrm{KL}}\left[p:{\left(pq\right)}_{\frac{1}{2}}^{M}\right],D_{\mathrm{KL}}\left[q:{\left(pq\right)}_{\frac{1}{2}}^{M}\right]\right),$$

where ${\left(pq\right)}_{\alpha }^{M}$ denotes the statistical weighted *M*-mixture:

(24) $${\left(pq\right)}_{\alpha }^{M}:=\frac{M_{\alpha }\left(p\left(x\right),q\left(x\right)\right)}{\int_{X}M_{\alpha }\left(p\left(x\right),q\left(x\right)\right)d\mu \left(x\right)}.$$

Notice that, when M=N=A (the arithmetic mean), Equation (23) of the (A,A)-JSD reduces to the ordinary JSD of Equation (5). When the means *M* and *N* are symmetric, the (M,N)-JSD is symmetric.

In general, a weighted mean $M_{\alpha }\left(a,b\right)$ for any α∈[0,1] shall satisfy the in-betweeness property [26] (i.e., a mean should be contained inside its extrema):

(25) $$\min\left\{a,b\right\}\leq M_{\alpha }\left(a,b\right)\leq \max\left\{a,b\right\}.$$

The three Pythagorean means defined for positive scalars a>0 and b>0 are classic examples of means:

- The arithmetic mean $A\left(a,b\right)=\frac{a+b}{2}$,
- the geometric mean $G\left(a,b\right)=\sqrt{ab}$, and
- the harmonic mean $H\left(a,b\right)=\frac{2ab}{a+b}$.

These Pythagorean means may be interpreted as special instances of another parametric family of means: The power means

(26) $$P_{\alpha }\left(a,b\right):={\left(\frac{a^{\alpha }+b^{\alpha }}{2}\right)}^{\frac{1}{\alpha }},$$

defined for α∈R\{0} (also called Hölder means). The power means can be extended to the full range α∈R by using the property that $\lim_{\alpha \to 0}P_{\alpha }\left(a,b\right)=G\left(a,b\right)$. The power means are homogeneous means: $P_{\alpha }\left(\lambda a,\lambda b\right)=\lambda P_{\alpha }\left(a,b\right)$ for any λ>0. We refer to the handbook of means [27] to obtain definitions and principles of other means beyond these power means.

A weighted mean (also called barycenter) can be built from a non-weighted mean M(a,b) (i.e., $\alpha =\frac{1}{2}$) by using the dyadic expansion of the real weight α∈[0,1], see [28]. That is, we can define the weighted mean M(p,q;w,1−w) for $w=\frac{i}{2^{k}}$ with $i\in \{0,\ldots ,2^{k}\}$ and *k* an integer. For example, consider a symmetric mean M(p,q)=M(q,p). Subsequently, we get the following weighted means when k=3:

$$\begin{array}{ccc} M\left(p,q;\frac{0}{8}=0,\frac{8}{8}=1\right) & = & q\\ M\left(p,q;\frac{1}{8},\frac{7}{8}\right) & = & M(M(M(p,q),q),q)\\ M\left(p,q;\frac{2}{8}=\frac{1}{4},\frac{6}{8}=\frac{3}{4}\right) & = & M(M(p,q),q)\\ M\left(p,q;\frac{3}{8},\frac{5}{8}\right) & = & M(M(M(p,q),p),q)\\ M\left(p,q;\frac{4}{8}=\frac{1}{2},\frac{4}{8}=\frac{1}{2}\right) & = & M(p,q)\\ M\left(p,q;\frac{5}{8},\frac{3}{8}\right) & = & M(M(M(p,q),q),p)\\ M\left(p,q;\frac{6}{8}=\frac{3}{4},\frac{2}{8}=\frac{1}{4}\right) & = & M(M(p,q),p)\\ M\left(p,q;\frac{7}{8},\frac{1}{8}\right) & = & M(M(M(p,q),p),p)\\ M\left(p,q;\frac{8}{8}=1,\frac{0}{8}=0\right) & = & p \end{array}$$

Let $w=\sum_{i=1}^{\infty }\frac{d_{i}}{2^{i}}$ be the unique dyadic expansion of the real number w∈(0,1), where the $d_{i}$’s are binary digits (i.e., $d_{i}\in \left\{0,1\right\}$). We define the weighted mean M(x,y;w,1−w) of two positive reals *p* and *q* for a real weight w∈(0,1) as

(27) $$M\left(x,y;w,1-w\right):=\lim_{n\to \infty }M\left(x,y;\sum_{i=1}^{n}\frac{d_{i}}{2^{i}},1-\sum_{i=1}^{n}\frac{d_{i}}{2^{i}}\right).$$

Choosing the abstract mean *M* in accordance with the family $R=\{p_{\theta }\;:\;\theta \in \Theta \}$ of the densities allows one to obtain closed-form formula for the (M,N)-JSDs that rely on definite integral calculations [23]. For example, the JSD between two Gaussian densities does not admit a closed-form formula because of the log-sum integral, but the (G,N)-JSD admits a closed-form formula when using geometric statistical mixtures (i.e., when M=G). The calculus trick is to find a weighted mean $M_{\alpha }$, such that, for two densities $p_{\theta_{1}}$ and $p_{\theta_{2}}$, the weighted mean distribution $M_{\alpha }\left(p_{\theta_{1}}\left(x\right),p_{\theta_{2}}\left(x\right)\right)=\frac{p_{\theta_{1,2,\alpha }}\left(x\right)}{Z_{M_{\alpha }}\left(\theta_{1},\theta_{2}\right)}$, where $Z_{M_{\alpha }}\left(\theta_{1},\theta_{2}\right)$ is the normalizing coefficient and $p_{\theta_{1,2,\alpha }}\in R$. Thus, the integral calculation can be simply calculated as $\int M_{\alpha }\left(p_{\theta_{1}}\left(x\right),p_{\theta_{2}}\left(x\right)\right)d\mu \left(x\right)=\frac{1}{Z_{M_{\alpha }}\left(\theta_{1},\theta_{2}\right)}$ since $p_{\theta_{1,2,\alpha }}\left(x\right)$, and, therefore, $\int p_{\theta_{1,2,\alpha }}\left(x\right)d\mu \left(x\right)=1$. This trick has also been used in Bayesian hypothesis testing for upper bounding the probability of error between two densities of a parametric family of distributions by replacing the usual geometric mean (Section 11.7 of [6], page 375) by a more general quasi-arithmetic mean [29]. For example, the harmonic mean is well-suited to Cauchy distributions, and the power means to Student *t*-distributions [29].

As an application of these generalized JSDs, Deasy et al. [30] used the skewed geometric JSD (namely, the $\left(G_{\alpha },A_{1-\alpha }\right)$-JSD for α∈(0,1)), which admits a closed-form formula between normal densities [23], and showed how regularizing an optimization task with this G-JSD divergence improved reconstruction and generation of Variational AutoEncoders (VAEs).

More generally, instead of using the KLD, one can also use any arbitrary distance *D* to define its JS-symmetrization, as follows:

(28) $$D_{M,N}^{\mathrm{JS}}\left[p:q\right]:=N\left(D\left[p:{\left(pq\right)}_{\frac{1}{2}}^{M}\right],D\left[q:{\left(pq\right)}_{\frac{1}{2}}^{M}\right]\right).$$

These symmetrizations may further be skewed by using $M_{\alpha }$ and/or $N_{\beta }$ for α∈(0,1) and β∈(0,1), yielding the definition [23]:

(29) $$D_{M_{\alpha },N_{\beta }}^{\mathrm{JS}}\left[p:q\right]:=N_{\beta }\left(D\left[p:{\left(pq\right)}_{\alpha }^{M}\right],D\left[q:{\left(pq\right)}_{\alpha }^{M}\right]\right).$$

With these notations, the ordinary JSD is $D_{\mathrm{JS}}={D_{\mathrm{KL}}}_{A,A}^{\mathrm{JS}}$, the (A,A) JS-symmetrization of the KLD with respect to the arithmetic means M=A and N=A.

The JS-symmetrization can be interpreted as the $N_{\beta }$-Jeffreys’ symmetrization of a generalization of Lin’s α-skewed *K*-divergence [4]$D_{M_{\alpha }}^{K}\left[p:q\right]$:

(30) $$\begin{array}{ccc} D_{M_{\alpha },N_{\beta }}^{\mathrm{JS}}\left[p:q\right] & = & N_{\beta }\left(D_{M_{\alpha }}^{K}\left[p:q\right],D_{M_{\alpha }}^{K}\left[p:q\right]\right), \end{array}$$

(31) $$\begin{array}{ccc} D_{M_{\alpha }}^{K}\left[p:q\right] & := & D\left[p:{\left(pq\right)}_{\alpha }^{M_{\alpha }}\right]. \end{array}$$

In this work, we consider symmetrizing an arbitrary distance *D* (including the KLD), generalizing the Jensen-Shannon divergence by using a variational formula for the JSD. Namely, we observe that the Jensen-Shannon divergence can also be defined as the following minimization problem:

(32) $$D_{\mathrm{JS}}\left[p,q\right]:=\min_{c\in D}\frac{1}{2}\left(D_{\mathrm{KL}}\left[p:c\right]+D_{\mathrm{KL}}\left[q:c\right]\right),$$

since the optimal density *c* is proven unique using the calculus of variation [1,31,32] and it corresponds to the mid density $\frac{p+q}{2}$, a statistical (arithmetic) mixture.

**Proof.**  Let $S\left(c\right)=D_{\mathrm{KL}}\left[p:c\right]+D_{\mathrm{KL}}\left[q:c\right]\geq 0$. We use the method of the Lagrange multipliers for the constrained optimization problem $\min_{c}S\left(c\right)$ such that ∫c(x)dμ(x)=1. Let us minimize $S\left(c\right)+\lambda \left(\int c(x)d\mu (x)-1\right)$. The density *c* realizing the minimum S(c) satisfies the Euler–Lagrange equation $\frac{\partial L}{\partial c}=0$, where $L\left(c\right):=p\log\frac{p}{c}+q\log\frac{q}{c}+\lambda c$ is the Lagrangian. That is, $-\frac{p}{c}-\frac{q}{c}+\lambda =0$ or, equivalently, $c=\frac{1}{\lambda }\left(p+q\right)$. Parameter λ is then evaluated from the constraint $\int_{X}c\left(x\right)d\mu \left(x\right)=1$: we get λ=2 since $\int_{X}\left(p\left(x\right)+q\left(x\right)\right)d\mu \left(x\right)=2$. Therefore, we find that $c\left(x\right)=\frac{p(x)+q(x)}{2}$, the mid density of p(x) and q(x). □

Considering Equation (32) instead of Equation (5) for defining the Jensen-Shannon divergence is interesting, because it allows one to consider a novel approach for generalizing the Jensen-Shannon divergence. This variational approach was first considered by Sibson [1] to define the α-information radius of a set of weighted distributions while using Rényi α-entropies that are based on Rényi principled α-means [33]. The α-information radius includes the Jensen-Shannon diversity index when α=1. Sibson’s work is our point of departure for generalizing the Jensen-Shannon divergence and proposing the Jensen-Shannon symmetrizations of arbitrary distances.

The paper is organized, as follows: in Section 2, we recall the rationale and definitions of the Rényi α-entropy and the Rényi α-divergence [33], and explain the information radius of Sibson [1], which includes, as a special case, the ordinary Jensen-Shannon divergence and that can be interpreted as generalized skew Bhattacharyya distances. We report, in Theorem 2, a closed-form formula for calculating the information radius of order α between two densities of an exponential family when $\frac{1}{\alpha }$ is an integer. It is noteworthy to point out that Sibson’s work (1969) includes, as a particular case of the information radius, a definition of the JSD, prior to the well-known reference paper of Lin [4] (1991). In Section 3, we present the JS-symmetrization variational definition that is based on a generalization of the information radius with a generic mean (Equation (88) and Definition 3). In Section 4, we constrain the mixture density to belong to a prescribed class of (parametric) probability densities, like an exponential family [2], and obtain a relative information radius generalizing information radius and related to the concept of information projections. Our Definition 5 generalizes the (relative) normal information radius of Sibson [1], who considered the multivariate normal family (Proposition 4). We illustrate this notion of relative information radius by calculating the density of an exponential family minimizing the reverse Kullback–Leibler divergence between a mixture of densities of that exponential family (Proposition 6). Moreover, we get a semi-closed-form formula for the Kullback–Leibler divergence between the densities of two different exponential families (Proposition 5), generalizing the Fenchel–Young divergence [34]. As an application of these relative variational JSDs, we touch upon the problems of clustering and quantization of probability densities in Section 4.2. Finally, we conclude by summarizing our contributions and discussing related works in Section 5.

## 2. Rényi Entropy and Divergence, and Sibson Information Radius

Rényi [33] investigated a generalization of the four axioms of Fadeev [35], yielding the unique Shannon entropy [20]. In doing so, Rényi replaced the ordinary weighted arithmetic mean by a more general class of averaging schemes. Namely, Rényi considered the weighted quasi-arithmetic means [36]. A weighted quasi-arithmetic mean can be induced by a strictly monotonous and continuous function *g*, as follows:

(33) $$M_{g}\left(x_{1},\ldots ,x_{n};w_{1},\ldots ,w_{n}\right):=g^{-1}\left(\sum_{i=1}^{n}w_{i}g\left(x_{i}\right)\right),$$

where the $x_{i}$’s and the $w_{i}$’s are positive (the weights are normalized, so that $\sum_{i=1}^{n}w_{i}=1$). Because $M_{g}=M_{-g}$, we may assume without loss of generality that *g* is a strictly increasing and continuous function. The quasi-arithmetic means were investigated independently by Kolmogorov [36], Nagumo [37], and de Finetti [38].

For example, the power means $P_{\alpha }\left(a,b\right)={\left(\frac{a^{\alpha }+b^{\alpha }}{2}\right)}^{\frac{1}{\alpha }}$ introduced earlier are quasi-arithmetic means for the generator $g_{\alpha }^{P}\left(u\right):=u^{\alpha }$:

(34) $$P_{\alpha }\left(a,b\right)=M_{g_{\alpha }^{P}}\left(a,b;\frac{1}{2},\frac{1}{2}\right).$$

Rényi proved that, among the class of weighted quasi-arithmetic means, only the means induced by the family of functions

(35) $$\begin{array}{ccc} g_{\alpha }\left(u\right) & := & 2^{(\alpha -1)u}, \end{array}$$

(36) $$\begin{array}{ccc} g_{\alpha }^{-1}\left(v\right) & := & \frac{1}{\alpha -1}\log_{2}v, \end{array}$$

for α>0 and α≠1 yield a proper generalization of Shannon entropy, nowadays called the Rényi α-entropy. The Rényi α-mean is

(37) $$\begin{array}{ccc} M_{\alpha }^{R}\left(x_{1},\ldots ,x_{n};w_{1},\ldots ,w_{n}\right) & = & M_{g_{\alpha }}\left(x_{1},\ldots ,x_{n};w_{1},\ldots ,w_{n}\right), \end{array}$$

(38) $$\begin{array}{ccc} & = & \frac{1}{\alpha -1}\log_{2}\left(\sum_{i=1}^{n}w_{i}2^{\left(\alpha -1\right)x_{i}}\right). \end{array}$$

The Rényi α-means $M_{\alpha }^{R}$ are not power means: They are not homogeneous means [31]. Let $M_{\alpha }^{R}\left(p,q\right)=M_{\alpha }^{R}\left(p,q;\frac{1}{2},\frac{1}{2}\right)=\frac{1}{\alpha -1}\log_{2}\frac{2^{(\alpha -1)p}+2^{(\alpha -1)q}}{2}$. Subsequently, we have $\lim_{\alpha \to \infty }$$M_{\alpha }^{R}\left(p,q\right)=\max\left\{p,q\right\}$ and $\lim_{\alpha \to 1}M_{\alpha }^{R}\left(p,q\right)=A\left(p,q\right)=\frac{p+q}{2}$. Indeed, we have

$$\begin{array}{ccc} M_{\alpha }^{R}\left(p,q\right) & = & \frac{1}{\alpha -1}\log_{2}\frac{2^{(\alpha -1)p}+2^{(\alpha -1)q}}{2},\\ & = & \frac{1}{\alpha -1}\log_{2}\frac{e^{(\alpha -1)p\log2}+e^{(\alpha -1)q\log2}}{2},\\ & \approx_{\alpha \to 1} & \frac{1}{\alpha -1}\log_{2}\left(1+\left(\alpha -1\right)\frac{p+q}{2}\log2\right),\\ & \approx_{\alpha \to 1} & \frac{1}{\alpha -1}\frac{1}{\log2}\left(\alpha -1\right)\frac{p+q}{2}\log2,\\ & \approx_{\alpha \to 1} & \frac{p+q}{2}=A\left(p,q\right), \end{array}$$

using the following first-order approximations: $e^{x}\approx_{x\to 0}=1+x$ and $\log\left(1+x\right)\approx_{x\to 0}=x$.

To obtain an intuition of the Rényi entropy, we may consider generalized entropies derived from quasi-arithmetic means, as follows:

(39) $$h_{g}\left[p\right]:=-M_{g}\left(\log_{2}p_{1},\ldots ,\log_{2}p_{n};p_{1},\ldots ,p_{n}\right).$$

When g(u)=u, we recover Shannon entropy. When $g_{2}\left(u\right)=2^{u}$, we get $h_{g_{2}}\left[p\right]=-\log_{2}\sum_{i}p_{i}^{2}$, called the collision entropy, since $-\log\mathrm{Pr}\left[X_{1}=X_{2}\right]=h_{g_{2}}\left[p\right]$, when $X_{1}$ and $X_{2}$ are independent and identically distributed random variables with $X_{1}∼p$ and $X_{2}∼p$. When $g\left(u\right)=g_{\alpha }\left(u\right)=2^{(\alpha -1)u}$, we get

(40) $$\begin{array}{ccc} h_{g_{\alpha }}\left[p\right] & = & -\frac{1}{\alpha -1}\log_{2}\left(\sum_{i}p_{i}2^{\left(\alpha -1\right)\log_{2}p_{i}}\right), \end{array}$$

(41) $$\begin{array}{ccc} & = & \frac{1}{1-\alpha }\log_{2}\sum_{i}p_{i}p_{i}^{\alpha -1}=\frac{1}{1-\alpha }\log_{2}\sum_{i}p_{i}^{\alpha }. \end{array}$$

The formula of Equation (41) is the discrete Rényi α-entropy [33], which can be defined more generally on a measure space (X,F,μ), as follows:

(42) $$h_{\alpha }^{R}\left[p\right]:=\frac{1}{1-\alpha }\log\left(\int_{X}p^{\alpha }\left(x\right)d\mu \left(x\right)\right),\;\alpha \in \left(0,1\right)\cup \left(1,\infty \right).$$

In the limit case α→1, the Rényi α-entropy converges to Shannon entropy: $\lim_{\alpha \to 1}h_{\alpha }^{R}\left[p\right]=h\left[p\right]$. Rényi α-entropies are non-increasing with respect to increasing α: $h_{\alpha }^{R}\left[p\right]\geq h_{\alpha^{\prime }}^{R}\left[p\right]$ for $\alpha <\alpha^{\prime }$. In the discrete case (i.e., counting measure μ on a finite alphabet X), we can further define $h_{0}\left[p\right]=\log\left|X\right|$ for α=0 (also called max-entropy or Hartley entropy). The Rényi +∞-entropy

$$h_{+\infty }\left[p\right]=-\log\max_{x\in X}p\left(x\right)$$

is also called the min-entropy, since the sequence $h_{\alpha }$ is non-increasing with respect to increasing α.

Similarly, Rényi obtained the α-divergences for α>0 and α≠1 (originally called information gain of order α):

(43) $$D_{\alpha }^{R}\left[p:q\right]:=\frac{1}{\alpha -1}\log_{2}\left(\int_{X}p{\left(x\right)}^{\alpha }q{\left(x\right)}^{1-\alpha }d\mu \left(x\right)\right),$$

generalizing the Kullback–Leibler divergence, since $\lim_{\alpha \to 1}D_{\alpha }^{R}\left[p:q\right]=D_{\mathrm{KL}}\left[p:q\right]$. Rényi α-divergences are non-decreasing with respect to increasing α [39]: $D_{\alpha }^{R}\left[p:q\right]\leq D_{\alpha^{\prime }}^{R}\left[p:q\right]$ for $\alpha^{\prime }\geq \alpha$.

Sibson (Robin Sibson (1944–2017) is also renown for inventing the natural neighbour interpolation [40]) [1] considered both the Rényi α-divergence [33]$D_{\alpha }^{R}$ and the Rényi α-weighted mean $M_{\alpha }^{R}:=M_{g_{\alpha }}$ to define the information radius $R_{\alpha }$ of order α of a weighted set $P={\left\{\left(w_{i},p_{i}\right)\right\}}_{i=1}^{n}$ of densities $p_{i}$’s as the following minimization problem:

(44) $$R_{\alpha }\left(P\right):=\min_{c\in D}R_{\alpha }\left(P,c\right),$$

where

(45) $$R_{\alpha }\left(P,c\right):=M_{\alpha }^{R}\left(D_{\alpha }^{R}\left[p_{1}:c\right],\ldots ,D_{\alpha }^{R}\left[p_{n}:c\right];w_{1},\ldots ,w_{n}\right).$$

The Rényi α-weighted mean $M_{\alpha }^{R}$ can be rewritten as

(46) $$\begin{array}{ccc} M_{\alpha }^{R}\left(x_{1},\ldots ,x_{n};w_{1},\ldots ,w_{n}\right) & = & \frac{1}{\alpha -1}\mathrm{LSE}\left(\left(\alpha -1\right)x_{1}\log2+\log w_{1},\ldots ,\left(\alpha -1\right)x_{i}\log2+\log w_{i}\right), \end{array}$$

where function $\mathrm{LSE}\left(a_{1},\ldots ,a_{n}\right):=\log\left(\sum_{i=1}^{n}e^{a_{i}}\right)$ denotes the log-sum-exp (convex) function [41,42].

Notice that $2^{\left(\alpha -1\right)D_{\alpha }^{R}\left[p:q\right]}=\int_{X}p{\left(x\right)}^{\alpha }q{\left(x\right)}^{1-\alpha }d\mu \left(x\right)$, the Bhattacharyya α-coefficient [12] (also called Chernoff α-coefficient [43,44]):

(47) $$C_{\mathrm{Bhat},\alpha }\left[p:q\right]:=\int_{X}p{\left(x\right)}^{\alpha }q{\left(x\right)}^{1-\alpha }d\mu \left(x\right).$$

Thus, we have

(48) $$R_{\alpha }\left(P,c\right)=\frac{1}{\alpha -1}\log_{2}\left(\sum w_{i}C_{\mathrm{Bhat},\alpha }\left[p_{i}:c\right]\right).$$

The ordinary Bhattacharyya coefficient is obtained for $\alpha =\frac{1}{2}$: $C_{\mathrm{Bhat}}\left[p:q\right]:=\int_{X}\sqrt{p(x)}$$\sqrt{q(x)}d\mu \left(x\right)$.

Sibson [1] also considered the limit case α→∞ when defining the information radius:

(49) $$D_{\infty }^{R}\left[p:q\right]:=\log_{2}\underset{x\in X}{\sup}\frac{p(x)}{q(x)}.$$

Sibson reported the following theorem in his information radius study [1]:

**Theorem** **1** (Theorem 2.2 and Corollary 2.3 of [1])**.** The optimal density $c_{\alpha }^{*}=\arg\min_{c\in D}R_{\alpha }$(P,c) is unique, and we have:

$$\begin{array}{ccc} c_{1}^{*}\left(x\right)=\sum_{i}w_{i}p_{i}\left(x\right), & R_{1}\left(P\right)=R_{1}\left(P,c_{1}^{*}\right)=\int_{X}\sum_{i}w_{i}p_{i}\log_{2}\frac{p_{i}}{\sum_{j}w_{j}p_{j}\left(x\right)}d\mu \left(x\right), & \\ c_{\alpha }^{*}\left(x\right)=\frac{{\left(\sum_{i}w_{i}p_{i}{\left(x\right)}^{\alpha }\right)}^{\frac{1}{\alpha }}}{\int_{X}{\left(\sum_{i}w_{i}p_{i}{\left(x\right)}^{\alpha }\right)}^{\frac{1}{\alpha }}d\mu \left(x\right)}, & R_{\alpha }\left(P\right)=R_{\alpha }\left(P,c_{\alpha }^{*}\right)=\frac{1}{\alpha -1}\log_{2}{\left(\int_{X}{\left(\sum_{i}w_{i}p_{i}{\left(x\right)}^{\alpha }\right)}^{\frac{1}{\alpha }}d\mu \left(x\right)\right)}^{\alpha }, & \\ & \alpha \in (0,1)\cup (1,\infty )\\ c_{\infty }^{*}\left(x\right)=\frac{\max_{i}p_{i}\left(x\right)}{\int_{X}\left(\max_{i}p_{i}\left(x\right)\right)d\mu \left(x\right)}, & R_{\infty }\left(P\right)=R_{\infty }\left(P,c_{\infty }^{*}\right)=\log_{2}\int_{X}\left(\max_{i}p_{i}\left(x\right)\right)d\mu \left(x\right), \end{array}$$

Observe that $R_{\infty }\left(P\right)$ does not depend on the (positive) weights.

The proof follows from the following decomposition of the information radius:

**Proposition** **1.** *We have:*

(50) $$R_{\alpha }\left(P,c\right)-R_{\alpha }\left(P,c_{\alpha }^{*}\right)=D_{\alpha }^{R}\left(c_{\alpha }^{*},c\right)\geq 0.$$

Because the proof is omitted in [1], we report it here:

**Proof.**  Let $\Delta \left(c,c_{\alpha }^{*}\right):=R_{\alpha }\left(P,c\right)-R_{\alpha }\left(P,c_{\alpha }^{*}\right)$. We handle the three cases, depending on the α values:

- Case α∈(0,1)∪(1,∞): Let $P_{\alpha }\left(P\right)\left(x\right):={\left(\sum_{i}w_{i}p_{i}{\left(x\right)}^{\alpha }\right)}^{\frac{1}{\alpha }}$. We have ${\left(c_{\alpha }^{*}\left(x\right)\right)}^{\alpha }=\frac{\sum_{i}w_{i}p_{i}{\left(x\right)}^{\alpha }}{{\left(\int P_{\alpha }\left(P\right)\left(x\right)d\mu \left(x\right)\right)}^{\alpha }}$. We obtain
  (51) $$\begin{array}{ccc} \Delta (c,c_{\alpha }^{*}) & = & \frac{1}{\alpha -1}\log_{2}\left(\sum_{i}w_{i}\int p_{i}{\left(x\right)}^{\alpha }c{\left(x\right)}^{1-\alpha }d\mu \left(x\right)\right)-\frac{1}{\alpha -1}\log_{2}{\left(\int P_{\alpha }\left(P\right)\left(x\right)d\mu \left(x\right)\right)}^{\alpha }, \end{array}$$
  (52) $$\begin{array}{ccc} & = & \frac{1}{\alpha -1}\log_{2}\frac{\sum_{i}w_{i}\int p_{i}{\left(x\right)}^{\alpha }c{\left(x\right)}^{1-\alpha }d\mu }{{\left(\int P_{\alpha }\left(P\right)\left(x\right)d\mu \left(x\right)\right)}^{\alpha }}, \end{array}$$
  (53) $$\begin{array}{ccc} & = & \frac{1}{\alpha -1}\log_{2}\frac{\int \left(\sum_{i}w_{i}p_{i}{\left(x\right)}^{\alpha }\right)c{\left(x\right)}^{1-\alpha }}{{\left(\int P_{\alpha }\left(P\right)\left(x\right)d\mu \left(x\right)\right)}^{\alpha }}d\mu \left(x\right), \end{array}$$
  (54) $$\begin{array}{ccc} & = & \frac{1}{\alpha -1}\log_{2}\int {\left(c_{\alpha }^{*}\left(x\right)\right)}^{\alpha }c{\left(x\right)}^{1-\alpha }d\mu \left(x\right), \end{array}$$
  (55) $$\begin{array}{ccc} & := & D_{\alpha }^{R}\left(c_{\alpha }^{*},c\right). \end{array}$$
- Case α=1: we have $\Delta \left(c,c_{1}^{*}\right):=R_{1}\left(P,c\right)-R_{1}\left(P,c_{1}^{*}\right)$ with $c_{1}^{*}=\sum_{i}w_{i}p_{i}$. Because $R_{1}\left(P,c\right)=\sum_{i}w_{i}D_{\mathrm{KL}}\left[p_{i}:c\right]$, we have
  (56) $$\begin{array}{ccc} R_{1}\left(P,c\right) & = & \sum_{i}w_{i}h\left[p_{i}:c\right]-w_{i}h\left[p_{i}\right], \end{array}$$
  (57) $$\begin{array}{ccc} & = & h\left[\sum_{i}w_{i}p_{i}:c\right]-\sum_{i}w_{i}h\left[p_{i}\right], \end{array}$$
  (58) $$\begin{array}{ccc} & = & h\left[c_{1}^{*}:c\right]-\sum_{i}w_{i}h\left[p_{i}\right]. \end{array}$$
  It follows that
  (59) $$\begin{array}{ccc} \Delta (c,c_{1}^{*}) & = & h\left[c_{1}^{*}:c\right]-\sum_{i}w_{i}h\left[p_{i}\right]-\left(h\left[c_{1}^{*}:c_{1}^{*}\right]-\sum_{i}w_{i}h\left[p_{i}\right]\right), \end{array}$$
  (60) $$\begin{array}{ccc} & = & h^{[}c_{1}^{*}:c]-h\left[c_{1}^{*}\right], \end{array}$$
  (61) $$\begin{array}{ccc} & = & D_{\mathrm{KL}}\left[c_{1}^{*}:c\right]=D_{1}^{R}\left[c_{1}^{*}:c\right]. \end{array}$$
- Case α=∞: we have $c_{\infty }^{*}=\frac{\max_{i}p_{i}\left(x\right)}{\int (\max_{i}p_{i}\left(x\right))d\mu \left(x\right)}$, $R_{\infty }\left(P,c_{\infty }^{*}\right)=\log_{2}\int \left(\max_{i}p_{i}\left(x\right)\right)d\mu \left(x\right)$, and $D_{\infty }^{R}\left[p:q\right]=\log_{2}\sup_{x}\frac{p(x)}{q(x)}$. We have $R_{\infty }\left(P,c\right)=\log_{2}\sup_{x}\frac{p_{i}\left(x\right)}{c(x)}$ Thus, $\Delta \left(c,c_{\alpha }^{*}\right):=R_{\infty }\left(P,c\right)-R_{\infty }\left(P,c_{\infty }^{*}\right)=\log_{2}\sup_{x}\frac{c_{\infty }^{*}\left(x\right)}{c(x)}=D_{\infty }^{R}\left[c_{\infty }^{*}:c\right]$.

 □

It follows that

$$\min_{c}R_{\alpha }\left(P,c\right)=\min_{c}R_{\alpha }\left(P,c_{\alpha }^{*}\right)+D_{\alpha }^{R}\left(c_{\alpha }^{*},c\right)\equiv \min_{c}D_{\alpha }^{R}\left(c_{\alpha }^{*},c\right)\geq 0.$$

Thus we have $c=c_{\alpha }^{*}$ since $D_{\alpha }^{R}\left(c_{\alpha }^{*},c\right)$ is minimized for $c=c_{\alpha }^{*}$.

Notice that $c_{\infty }^{*}\left(x\right)=\frac{\max\{p_{1}\left(x\right),\ldots ,p_{n}\left(x\right)\}}{\int_{X}\left(\max_{i}p_{i}\left(x\right)\right)d\mu \left(x\right)}$ is the upper envelope of the densities $p_{i}\left(x\right)$’s normalized to be a density. Provided that the densities $p_{i}$’s intersect pairwise in at most *s* locations (i.e., $|\{p_{i}\left(x\right)\cap p_{j}\left(x\right)\}|\leq s$ for i≠j), we can efficiently compute this upper envelope using an output-sensitive algorithm [45] of computational geometry.

When the point set is $P=\left\{\left(\frac{1}{2},p\right),\left(\frac{1}{2},q\right)\right\}$ with $w_{1}=w_{2}=\frac{1}{2}$, the information radius defines a (2-point) symmetric distance, as follows:

$$\begin{array}{cc} R_{1}\left(p,q\right)=\frac{1}{2}\int_{X}p\left(x\right)\log_{2}\frac{2p}{p(x)+q(x)}d\mu \left(x\right)+\frac{1}{2}\int_{X}q\left(x\right)\log_{2}\frac{2q(x)}{p(x)+q(x)}d\mu \left(x\right), & \alpha =1\\ R_{\alpha }\left(p,q\right)=\frac{\alpha }{\alpha -1}\log_{2}\int_{X}{\left(\frac{p{\left(x\right)}^{\alpha }+q{\left(x\right)}^{\alpha }}{2}\right)}^{\frac{1}{\alpha }}d\mu \left(x\right)=\frac{\alpha }{\alpha -1}\log_{2}\int_{X}P_{\alpha }\left(p\left(x\right),q\left(x\right)\right)d\mu \left(x\right), & \alpha \in (0,1)\cup (1,\infty )\\ R_{\infty }\left(p,q\right)=\log_{2}\int_{X}\max\left\{p\left(x\right),q\left(x\right)\right\}d\mu \left(x\right), & \alpha =\infty . \end{array}$$

This family of symmetric divergences may be called the Sibson’s α-divergences, and the Jensen-Shannon divergence is interpreted as a limit case when α→1. Notice that, since we have $\lim_{\alpha \to \infty }P_{\alpha }\left(p,q\right)=\max\left\{p,q\right\}$ and $\lim_{\alpha \to \infty }\frac{\alpha }{\alpha -1}=1$, we have $\lim_{\alpha \to \infty }R_{\alpha }\left(p,q\right)=R_{\infty }\left(p,q\right)$. Notice that, for α=1, the integral and logarithm operations are swapped as compared to $R_{\alpha }$ for α∈(0,1)∪(1,∞).

**Theorem** **2.** *When $\alpha =\frac{1}{k}$ for an integer k≥2, the Sibson α-divergences between two densities $p_{\theta_{1}}$ and $p_{\theta_{2}}$ of an exponential family $\left\{p_{\theta }\;:\;\theta \in \Theta \right\}$ with cumulant function F(θ) is available in closed form:*

Rα(pθ1,pθ2)=−1k−1log212k∑i=0kkiexpFikθ1+1−ikθ2−ikF(θ1)+1−ikF(θ2).

**Proof.**  Let $p=p_{\theta_{1}}$ and $q=p_{\theta_{2}}$ be two densities of an exponential family [2] with cumulant function F(θ) and natural parameter space Θ. Without a loss of generality, we may consider a natural exponential family [2] with densities written canonically as $p_{\theta }\left(x\right)=\exp\left(x^{⊤}\theta -F\left(\theta \right)\right)$ for θ∈Θ. It can be shown that the cumulant function $F\left(\theta \right)=\log\int_{X}\exp\left(x^{⊤}\theta \right)d\mu \left(x\right)$ is strictly convex and analytic on the open convex natural parameter space Θ [2].

When $\alpha =\frac{1}{2}$ (i.e., k=2), we have:

(62) $$\begin{array}{ccc} R_{\frac{1}{2}}\left(p,q\right) & = & -\log_{2}\int_{X}{\left(\frac{\sqrt{p(x)}+\sqrt{q(x)}}{2}\right)}^{2}d\mu \left(x\right), \end{array}$$

(63) $$\begin{array}{ccc} & = & -\log_{2}\left(\frac{1}{2}+\frac{1}{2}\int_{X}\sqrt{p(x)}\sqrt{q(x)}d\mu \left(x\right)\right), \end{array}$$

(64) $$\begin{array}{ccc} & = & -\log_{2}\left(\frac{1}{2}+\frac{1}{2}C_{\mathrm{Bhat}}\left[p:q\right]\right)\geq 0, \end{array}$$

where $C_{\mathrm{Bhat}}\left[p:q\right]:=\int_{X}\sqrt{p(x)}\sqrt{q(x)}d\mu \left(x\right)$ is the Bhattacharyya coefficient (with $0\leq C_{\mathrm{Bhat}}\left[p:q\right]\leq 1$). Using Theorem 3 of [12], we have

$$C_{\mathrm{Bhat}}\left[p_{\theta_{1}},p_{\theta_{2}}\right]=\exp\left(F\left(\frac{\theta_{p}+\theta_{q}}{2}\right)-\frac{F\left(\theta_{p}\right)+F\left(\theta_{q}\right)}{2}\right),$$

so that we obtain the following closed-form formula:

$$R_{\frac{1}{2}}\left(p_{\theta_{1}},p_{\theta_{2}}\right)=-\log_{2}\left(\frac{1}{2}+\frac{1}{2}\exp\left(F\left(\frac{\theta_{p}+\theta_{q}}{2}\right)-\frac{F\left(\theta_{p}\right)+F\left(\theta_{q}\right)}{2}\right)\right)\geq 0,$$

Now, assume that $k=\frac{1}{\alpha }\geq 2$ is an arbitrary integer, and let us apply the binomial expansion for $P_{\alpha }\left(p_{\theta_{1}},p_{\theta_{2}}\right)$ in the spirit of [46,47]:

(65) $$\begin{array}{ccc} \int_{X}P_{\alpha }\left(p_{\theta_{1}}\left(x\right),p_{\theta_{2}}\left(x\right)\right)d\mu \left(x\right) & = & \int_{X}{\left(\frac{p_{\theta_{1}}{\left(x\right)}^{\frac{1}{k}}+p_{\theta_{2}}{\left(x\right)}^{\frac{1}{k}}}{2}\right)}^{k}d\mu \left(x\right), \end{array}$$

(66) =12k∑i=0kki∫Xpθ1(x)1kipθ2(x)1kk−idμ(x).

Let $I_{k,i}\left(\theta_{1},\theta_{2}\right):=\int_{X}{\left(p_{\theta_{1}}{\left(x\right)}^{\frac{1}{k}}\right)}^{i}{\left(p_{\theta_{2}}{\left(x\right)}^{\frac{1}{k}}\right)}^{k-i}d\mu \left(x\right)$. Because $\frac{i}{k}\theta_{1}+\frac{k-i}{k}\theta_{2}=\theta_{2}+\frac{i}{k}\left(\theta_{1}-\theta_{2}\right)\in \Theta$ for i∈{0,…,k}, we get by following the calculation steps in [12]:

$$I_{k,i}\left(\theta_{1},\theta_{2}\right):=\exp\left(F\left(\frac{i}{k}\theta_{1}+\left(1-\frac{i}{k}\right)\theta_{2}\right)-\left(\frac{i}{k}F\left(\theta_{1}\right)+\left(1-\frac{i}{k}\right)F\left(\theta_{2}\right)\right)\right)<\infty .$$

Notice that $I_{2,1}=C_{\mathrm{Bhat}}\left[p_{\theta_{1}},p_{\theta_{2}}\right]$, and $I_{k,0}=I_{k,k}=1$.

Thus, we get the following closed-form formula:

(67) Rα(pθ1,pθ2)=−1k−1log212k∑i=0kkiexpFikθ1+1−ikθ2−ikF(θ1)+1−ikF(θ2).

 □

This closed-form formula applies, in particular, to the family {N(μ,Σ)} of (multivariate) normal distributions: In this case, the natural parameters θ are expressed using both a vector parameter component *v* and a matrix parameter component *M*:

(68) $$\theta =\left(v,M\right)=\left(\Sigma^{-1}m,-\frac{1}{2}\Sigma^{-1}\right),$$

and the cumulant function is:

(69) $$F_{N}\left(\theta \right)=\frac{d}{2}\log\pi -\frac{1}{2}\log\left|-2M\right|-\frac{1}{4}v^{⊤}M^{-1}v,$$

where |·| denotes the matrix determinant.

In general, the optimal density $c_{\alpha }^{*}=\arg\min_{c\in D}R_{\alpha }\left(P,c\right)$ yielding the information radius $R_{\alpha }\left(P\right)$ can be interpreted as a generalized centroid (extending the notion of Fréchet means [48]) with respect to $\left(M_{\alpha }^{R},D_{\alpha }^{R}\right)$, where a (M,D)-centroid is defined by:

**Definition** **1** ((M,D)-centroid)**.** *Let $P=\{\left(w_{1},p_{1}\right),\ldots ,\left(w_{n},p_{n}\right)\}$ be a normalized weighted parameter set, M a mean, and D a distance. Subsequently, the (M,D)-centroid is defined as*

$$c_{M,D}\left(P\right)=\arg\min_{c}M\left(D\left(p_{1}:c\right),\ldots ,D\left(p_{n}:c\right);w_{1},\ldots ,w_{n}\right).$$

Here, we give a general definition of the (M,D)-centroid for an arbitrary distance (not necessarily a symmetric nor metric distance). The parameter set can either be probability measures having densities with respect to a given measure μ or a set of vectors. In the first case, the distance *D* is called a statistical distance. When the densities belong to a parametric family of densities $P=\{p_{\theta }\;:\;\theta \in \Theta \}$, the statistical distance $D[p_{\theta_{1}}:p_{\theta_{2}}]$ amounts to a parameter distance: $D_{P}\left(\theta_{1}:\theta_{2}\right):=D\left[p_{\theta_{1}}:p_{\theta_{2}}\right]$. For example, when all of the densities $p_{i}$’s belong to a same natural exponential family [2]

$$P=\{p_{\theta }\left(x\right)=\exp\left(\theta^{⊤}t\left(x\right)-F\left(\theta \right)\right)\;:\;\theta \in \Theta \}$$

with cumulant function $F\left(\theta \right)=\log\int \exp(\theta^{⊤}t\left(x\right))d\mu \left(x\right)$ (i.e., $p_{i}=p_{\theta_{i}}$) and sufficient statistic vector t(x), we have $D_{\mathrm{KL}}\left[p_{\theta }:p_{\theta_{i}}\right]=B_{F}^{*}\left(\theta :\theta_{i}\right):=B_{F}\left(\theta_{i}:\theta \right)$, where $B_{F}^{*}$ denotes the reverse Bregman divergence (by parameter order swapping) the Bregman divergence [21]$B_{F}$ defined by

(70) $$B_{F}\left(\theta :\theta^{\prime }\right):=F\left(\theta \right)-F\left(\theta^{\prime }\right)-{\left(\theta -\theta^{\prime }\right)}^{⊤}\nabla F\left(\theta^{\prime }\right).$$

Thus, we have $D_{P}\left(\theta_{1}:\theta_{2}\right):=B_{F}^{*}\left(\theta_{1}:\theta_{2}\right)=D_{\mathrm{KL}}\left[p_{\theta_{1}}:p_{\theta_{2}}\right]$.

Let $V=\{\left(w_{1},\theta_{1}\right),\ldots ,\left(w_{n},\theta_{n}\right)\}$ be the parameter set corresponding to P. Define

(71) $$R_{F}\left(V,\theta \right):=\sum_{i=1}^{n}w_{i}B_{F}\left(\theta_{i}:\theta \right).$$

Subsequently, we have the equivalent decomposition of Proposition 1:

(72) $$R_{F}\left(V,\theta \right)-R_{F}\left(V,\theta^{*}\right)=B_{F}\left(\theta^{*}:\theta \right),$$

with $\theta^{*}=\bar{\theta }:=\sum_{i=1}^{n}w_{i}\theta_{i}$. (this decomposition is used to prove Proposition 1 of [21]). The quantity $R_{F}\left(V\right)=R_{F}\left(V,\theta^{*}\right)$ was termed the Bregman information [21,49]. The Bregman information generalizes the variance that was obtained when the Bregman divergence is the squared Euclidean distance. $R_{F}\left(V\right)$ could also be called Bregman information radius according to Sibson. Because $R_{F}\left(V\right)=\sum_{i=1}^{n}w_{i}D_{\mathrm{KL}}\left[p_{\bar{\theta }}:p_{\theta_{i}}\right]$, we can interpret the Bregman information as a Sibson’s information radius for densities of an exponential family with respect to the arithmetic mean $M_{1}^{R}=A$ and the reverse Kullback–Leibler divergence: $D_{\mathrm{KL}}^{*}\left[p:q\right]:=D_{\mathrm{KL}}\left[q:p\right]$. This observation yields us the JS-symmetrization of distances based on generalized information radii in Section 3.

More generally, we may consider the densities belonging to a deformed *q*-exponential family (see [10], page 85–89 and the monograph [50]). Deformed *q*-exponential families generalize the exponential families, and include the *q*-Gaussians [10]. A common way to measure the statistical distance between two densities of a *q*-exponential family is the *q*-divergence [10], which is related to Tsallis’ entropy [51]. We may also define another statistical divergence between two densities of a *q*-exponential family which amounts to Bregman divergence. For example, we refer to [52] for details concerning the family of Cauchy distributions, which are *q*-Gaussians for q=2.

Sibson proved that the information radii of any order are all upper bounded (Theorem 2.8 and Theorem 2.9 of [1]) as follows:

(73) $$\begin{array}{ccc} R_{1}\left(P\right) & \leq & \sum_{i}w_{i}\log_{2}\frac{1}{w_{j}}\leq \log_{2}n<\infty , \end{array}$$

(74) $$\begin{array}{ccc} R_{\alpha }\left(P\right) & \leq & \frac{\alpha }{\alpha -1}\log_{2}\left(\sum_{i}w_{i}^{\frac{1}{\alpha }}\right)\leq \log_{2}n<\infty ,\;\alpha \in \left(0,1\right)\cup \left(1,\infty \right) \end{array}$$

(75) $$\begin{array}{ccc} R_{\infty }\left(P\right) & \leq & \log_{2}n<\infty . \end{array}$$

We interpret Sibson’s upper bounds of Equations (73)–(75), as follows:

**Proposition** **2** (Information radius upper bound)**.** *The information radius of order α of a weighted set of distributions is upper bounded by the discrete Rényi entropy of order $\frac{1}{\alpha }$ of the weight distribution: $R_{\alpha }\left(P\right)\leq H_{\frac{1}{\alpha }}^{R}\left[w\right]$, where $H_{\alpha }^{R}\left[w\right]:=\frac{1}{1-\alpha }\log\left(\sum_{i}w_{i}^{\alpha }\right)$.*

## 3. JS-Symmetrization of Distances Based on Generalized Information Radius

Let us give the following definitions generalizing the information radius (i.e., Jensen-Shannon symmetrization of the distance when |P|=2) and the ordinary Jensen-Shannon divergence:

**Definition** **2** ((M,D)-information radius)**.** *Let M be a weighted mean and D a distance. Subsequently, the generalized information radius for a weighted set of points (e.g., vectors or densities) $\left(w_{1},p_{1}\right),\ldots ,\left(w_{n},p_{n}\right)$ is:*

$$R_{M,D}\left(P\right):=\min_{c\in D}M\left(D\left(p_{1}:c\right),\ldots ,D\left(p_{n}:c\right);w_{1},\ldots ,w_{n}\right).$$

Recall that we also defined the (M,D)-centroid in Definition 1 as follows:

$$c_{M,D}\left(P\right):=\arg\min_{c\in D}M\left(D\left(p_{1}:c\right),\ldots ,D\left(p_{n}:c\right);w_{1},\ldots ,w_{n}\right).$$

When M=A, we recover the notion of Fréchet mean [48]. Notice that, although the minimum $R_{M,D}\left(P\right)$ is unique, several generalized centroids $c_{M,D}\left(P\right)$ may potentially exist, depending on (M,D). In particular, Definition 2 and Definition 1 apply when *D* is a statistical distance, i.e., a distance between densities (Radon–Nikodym derivatives of corresponding probability measures with respect to a dominating measure μ).

The generalized information radius can be interpreted as a diversity index or an *n*-point distance. When n=2, we get the following (2-point) distances, which are considered as a generalization of the Jensen-Shannon divergence or Jensen-Shannon symmetrization:

**Definition** **3** (*M*-vJS symmetrization of *D*)**.** *Let M be a mean and D a statistical distance. Subsequently, the variational Jensen-Shannon symmetrization of D is defined by the formula of a generalized information radius:*

$$D_{M}^{\mathrm{vJS}}\left[p:q\right]:=\min_{c\in D}M\left(D[p:c],D[q:c]\right).$$

We use the acronym vJS to distinguish it with the JS-symmetrization reported in [23]:

$$D_{M}^{\mathrm{JS}}\left[p:q\right]=D_{M,A}^{\mathrm{JS}}\left[p:q\right]:=\frac{1}{2}\left(D\left[p:{\left(pq\right)}_{\frac{1}{2}}^{M}\right]+D\left[q:{\left(pq\right)}_{\frac{1}{2}}^{M}\right]\right).$$

We recover Sibson’s information radius $R_{\alpha }\left[p:q\right]$ induced by two densities *p* and *q* from Definition 3 as the *$M_{\alpha }^{R}$-vJS symmetrization of the Rényi divergence $D_{\alpha }^{R}$*. We have ${B_{F}}_{A}^{\mathrm{vJS}}$, which is the Bregman information [21]. Notice that we may skew these generalized JSDs by taking weighted mean $M_{\beta }$ instead of *M* for β∈(0,1), yielding the general definition:

**Definition** **4** (Skew $M_{\beta }$-vJS symmetrization of *D*)**.** *Let $M_{\beta }$ be a weighted mean and D a statistical distance. Subsequently, the variational skewed Jensen-Shannon symmetrization of D is defined by the formula of a generalized information radius:*

$$\begin{array}{c} D_{M_{\beta }}^{\mathrm{vJS}}\left[p:q\right]:=\min_{c\in D}M_{\beta }\left(D[p:c],D[q:c]\right) \end{array}$$

**Example** **1.** *For example, the skewed Jensen–Bregman divergence of Equation (20) can be interpreted as a Jensen-Shannon symmetrization of the Bregman divergence $B_{F}$ [12] since we have:*

(76) $$\begin{array}{ccc} {B_{F}}_{A_{\beta }}^{\mathrm{vJS}}\left(\theta_{1}:\theta_{2}\right) & = & \min_{\theta \in \Theta }A_{\beta }\left(B_{F}\left(\theta_{1}:\theta \right),B_{F}\left(\theta_{2}:\theta \right)\right), \end{array}$$

(77) $$\begin{array}{ccc} & = & \min_{\theta \in \Theta }\left(1-\beta \right)B_{F}\left(\theta_{1}:\theta \right)+\beta B_{F}\left(\theta_{2}:\theta \right), \end{array}$$

(78) $$\begin{array}{ccc} & = & \left(1-\beta \right)B_{F}\left(\theta_{1}:\left(1-\beta \right)\theta_{1}+\beta \theta_{2}\right)+\beta B_{F}\left(\theta_{2}:\left(1-\beta \right)\theta_{1}+\beta \theta_{2}\right), \end{array}$$

(79) $$\begin{array}{ccc} & =: & {\mathrm{JB}}_{F,\beta }\left(\theta_{1}:\theta_{2}\right). \end{array}$$

*Indeed, the Bregman barycenter $\arg\min_{\theta \in \Theta }\left(1-\beta \right)B_{F}\left(\theta_{1}:\theta \right)+B_{F}\left(\theta_{2}:\theta \right)$ is unique and it corresponds to $\theta =\left(1-\beta \right)\theta_{1}+\beta \theta_{2}$, see [21]. The skewed Jensen–Bregman divergence ${\mathrm{JB}}_{F,\beta }\left(\theta_{1}:\theta_{2}\right)$ can also be rewritten as an equivalent skewed Jensen divergence (see Equation (22)):*

(80) $$\begin{array}{ccc} {\mathrm{JB}}_{F,\beta }\left(\theta_{1}:\theta_{2}\right) & = & \left(1-\beta \right)B_{F}\left(\theta_{1}:\left(1-\beta \right)\theta_{1}+\beta \theta_{2}\right)+\beta B_{F}\left(\theta_{2}:\left(1-\beta \right)\theta_{1}+\beta \theta_{2}\right), \end{array}$$

(81) $$\begin{array}{ccc} & = & \left(1-\beta \right)F\left(\theta_{1}\right)+\beta F\left(\theta_{2}\right)-F\left(\left(1-\beta \right)\theta_{1}+\beta \theta_{2}\right), \end{array}$$

(82) $$\begin{array}{ccc} & =: & J_{F,\beta }\left(\theta_{1}:\theta_{2}\right). \end{array}$$

**Example** **2.** *Consider a conformal Bregman divergence [53] that is defined by*

(83) $$B_{F,\rho }\left(\theta_{1}:\theta_{2}\right)=\rho \left(\theta_{1}\right)B_{F}\left(\theta_{1}:\theta_{2}\right),$$

*where ρ(θ)>0 is a conformal factor. Subsequently, we have*

(84) $$\begin{array}{ccc} {B_{F,\rho }}_{A_{\beta }}^{\mathrm{vJS}}\left(\theta_{1}:\theta_{2}\right) & = & \min_{\theta \in \Theta }A_{\beta }\left(B_{F,\rho }\left(\theta_{1}:\theta \right),B_{F,\rho }\left(\theta_{2}:\theta \right)\right), \end{array}$$

(85) $$\begin{array}{ccc} & = & \min_{\theta \in \Theta }\left(1-\beta \right)B_{F,\rho }\left(\theta_{1}:\theta \right)+B_{F,\rho }\left(\theta_{2}:\theta \right), \end{array}$$

(86) $$\begin{array}{ccc} & = & \left(1-\beta \right)B_{F}\left(\theta_{1}:\gamma_{1}\theta_{1}+\gamma_{2}\theta_{2}\right)+\beta B_{F}\left(\theta_{2}:\gamma_{1}\theta_{1}+\gamma_{2}\theta_{2}\right), \end{array}$$

*where $\gamma_{1}=\frac{\left(1-\beta \right)\rho \left(\theta_{1}\right)}{\left(1-\beta \right)\rho \left(\theta_{1}\right)+\beta \rho \left(\theta_{2}\right)}$ and $\gamma_{2}=\frac{\beta \rho (\theta_{2})}{\left(1-\beta \right)\rho \left(\theta_{1}\right)+\beta \rho \left(\theta_{2}\right)}=1-\gamma_{1}$.*

Notice that this definition is implicit and it can be made explicit when the centroid $c^{*}\left(p,q\right)$ is unique:

(87) $$D_{M_{\beta }}^{\mathrm{vJS}}\left[p:q\right]=M_{\beta }\left(D\left[p:c^{*}\left(p,q\right)\right],D\left[q:c^{*}\left(p,q\right)\right]\right.$$

In particular, when $D=D_{\mathrm{KL}}$, the KLD, we obtain generalized skewed Jensen-Shannon divergences for $M_{\beta }$ a weighted mean with β∈(0,1):

(88) $$D_{\mathrm{vJS}}^{M_{\beta }}\left[p:q\right]:=\min_{c\in D}M_{\beta }\left(D_{\mathrm{KL}}\left[p:c\right],D_{\mathrm{KL}}\left[q:c\right]\right).$$

**Example** **3.** *Amari [31] obtained the $\left(A,D_{\alpha }\right)$-information radius and its corresponding unique centroid for $D_{\alpha }$, the α-divergence of information geometry [10] (page 67).*

**Example** **4.** *Brekelmans et al. [54] studied the geometric path ${\left(p_{1}p_{2}\right)}_{\beta }^{G}\left(x\right)\propto p_{1}^{1-\beta }\left(x\right)p_{2}^{\beta }\left(x\right)$ between two distributions $p_{1}$ and $p_{2}$ of D, where $G_{\beta }\left(a,b\right)=a^{1-\beta }b^{\beta }$ (with a,b>0) is the weighted geometric mean. They proved the variational formula:*

(89) $${\left(p_{1}p_{2}\right)}_{\beta }^{G}=\min_{c\in D}\left(1-\beta \right)D_{\mathrm{KL}}\left[c:p_{1}\right]+\beta D_{\mathrm{KL}}\left[c:p_{2}\right].$$

That is, ${\left(p_{1}p_{2}\right)}_{\beta }^{G}$ is a $G_{\beta }$-$D_{\mathrm{KL}}^{*}$ centroid, where $D_{\mathrm{KL}}^{*}$ is the reverse KLD. The corresponding $\left(G_{\beta },D_{\mathrm{KL}}^{*}\right)$-vJSD is studied is [23] and it is used in deep learning in [30].

*It is interesting to study the link between $\left(M_{\beta },D\right)$-variational Jensen-Shannon symmetrization of D and the $\left(M_{\alpha }^{\prime },N_{\beta }^{\prime }\right)$-JS symmetrization of [23]. In particular, the link between $M_{\beta }$ for averaging in the minimization and $M_{\alpha }^{\prime }$ the mean for generating abstract mixtures.*

*More generally, Brekelmans et al. [55] considered the α-divergences extended to positive measures (i.e., a separable divergence built as the different between a weighted arithmetic mean and a geometric mean [56]):*

(90) $$D_{\alpha }^{e}\left[p:q\right]:=\frac{4}{1-\alpha^{2}}\int_{X}\left(\frac{1-\alpha }{2}p\left(x\right)+\frac{1+\alpha }{2}q\left(x\right)-p^{\frac{1-\alpha }{2}}\left(x\right)q^{\frac{1+\alpha }{2}}\left(x\right)\right)d\mu \left(x\right)$$

*and proved that*

(91) $$c_{\beta }^{*}=\arg\min_{c\in D}\left\{\left(1-\beta \right)D_{\alpha }^{e}\left[p_{1}:c\right]+\beta D_{\alpha }^{e}\left[p_{2}:c\right]\right\}$$

is a density of a likelihood ratio q-exponential family: $c_{\beta }^{*}=\frac{p_{1}\left(x\right)}{Z_{\beta ,q}}\exp_{q}\left(\beta \log_{q}\frac{p_{2}\left(x\right)}{p_{1}\left(x\right)}\right)$ for $q=\frac{1+\alpha }{2}$. That is, $c_{\beta }^{*}$ is the $\left(A_{\beta },D_{\alpha }^{e}\right)$-generalized centroid, and the corresponding information radius is the variational JS symmetrization:

(92) $${D_{\alpha }^{e}}^{\mathrm{vJS}}\left[p_{1}:p_{2}\right]=\left(1-\beta \right)D_{\alpha }^{e}\left[p_{1}:c_{\beta }^{*}\right]+\beta D_{\alpha }^{e}\left[p_{2}:c_{\beta }^{*}\right]$$

**Example** **5.** *The q-divergence [57]$D_{q}$ between two densities of a q-exponential family amounts to a Bregman divergence [10,57]. Thus, $D_{q}^{\mathrm{vJS}}$ for M=A is a generalized information radius that amounts to a Bregman information.*

For the case α=∞ in Sibson’s information radius, we find that the information radius is related to the total variation:

**Proposition** **3** (Lemma 2.4 [1])**.** *:*

(93) $$D_{\infty }^{\mathrm{vJS},R}\left[p:q\right]=\log_{2}\left(1+D_{\mathrm{TV}}\left[p:q\right]\right),$$

*where $D_{\mathrm{TV}}$ denotes the total variation*

(94) $$D_{\mathrm{TV}}\left[p:q\right]=\frac{1}{2}\int_{X}\left|p\left(x\right)-q\left(x\right)\right|d\mu \left(x\right).$$

**Proof.**  Because $\max\left\{p\left(x\right),q\left(x\right)\right\}=\frac{p(x)+q(x)}{2}+\frac{1}{2}\left|q\left(x\right)-p\left(x\right)\right|$, it follows that we have:

$$\int_{X}\max\left\{p\left(x\right),q\left(x\right)\right\}d\mu \left(x\right)=1+D_{\mathrm{TV}}\left[p:q\right].$$

From Theorem 1, we have $R_{\infty }(\left\{\left(\frac{1}{2},p\right),\left(\frac{1}{2},q\right)\right)=\log_{2}\int_{X}\max\left\{p\left(x\right),q\left(x\right)\right\}d\mu \left(x\right)$ and, therefore, $R_{\infty }(\left\{\left(\frac{1}{2},p\right),\left(\frac{1}{2},q\right)\right)=\log_{2}\left(1+D_{\mathrm{TV}}\left[p:q\right]\right)$. □

Notice that, when $M=M_{g}$ is a quasi-arithmetic mean, we may consider the divergence $D_{g}\left[p:q\right]=g^{-1}\left(D\left[p:q\right)\right)$, so that the centroid of the $\left(M_{g},D_{g}\right)$-JS symmetrization is:

(95) $$\arg\min_{c}g^{-1}\left(\sum_{i=1}^{n}w_{i}D\left[p_{i}:c\right]\right)\equiv \arg\min_{c}\sum_{i=1}^{n}w_{i}D\left[p_{i}:c\right].$$

The generalized α-skewed Bhattacharyya divergence [29] can also be considered with respect to a weighted mean $M_{\alpha }$:

$$D_{\mathrm{Bhat},M_{\alpha }}\left[p:q\right]=-\log\int_{X}M_{\alpha }\left(p\left(x\right),q\left(x\right)\right)d\mu \left(x\right).$$

In particular, when $M_{\alpha }$ is a quasi-arithmetic weighted mean that is induced by a strictly continuous and monotone function *g*, we have

$$D_{\mathrm{Bhat},g,\alpha }\left[p:q\right]:=-\log\int_{X}M_{g}\left(p\left(x\right),q\left(x\right);\alpha \right)d\mu \left(x\right)=:D_{\mathrm{Bhat},{\left(M_{g}\right)}_{\alpha }}\left[p:q\right].$$

Because $\min\left\{p\left(x\right),q\left(x\right)\right\}\leq M_{g}\left(p\left(x\right),q\left(x\right);\alpha \right)\leq \max\left\{p\left(x\right),q\left(x\right)\right\}$, $\min\left\{a,b\right\}=\frac{a+b}{2}-\frac{\left|b-a\right|}{2}$ and $\max\left\{a,b\right\}=\frac{a+b}{2}+\frac{\left|b-a\right|}{2}$, we deduce that we have:

(96) $$0\leq 1-D_{\mathrm{TV}}\left[p,q\right]\leq \int_{X}M_{g}\left(p\left(x\right),q\left(x\right);\alpha \right)d\mu \left(x\right)\leq 1+D_{\mathrm{TV}}\left[p,q\right]\leq 2.$$

The information radius of Sibson for α∈(0,1)∪(1,∞) may be interpreted as generalized scaled α-skewed Bhattacharyya divergences with respect to the power means $P_{\alpha }$, since we have $R_{\alpha }\left(p,q\right)=\frac{\alpha }{\alpha -1}\log_{2}\int_{X}P_{\alpha }\left(p\left(x\right),q\left(x\right);\alpha \right)d\mu \left(x\right)=\frac{\alpha }{1-\alpha }D_{\mathrm{Bhat},P_{\alpha }}\left[p:q\right]$.

## 4. Relative Information Radius and Relative Jensen-Shannon Symmetrizations of Distances

### 4.1. Relative Information Radius

In this section, instead of considering the full space of densities D on (X,F,μ) for performing the variational optimization of the information radius, we rather consider a subfamily of (parametric) densities R⊂D. Subsequently, we define accordingly the R-relative Jensen-Shannon divergence (R-JSD for short) as

(97) $$D_{\mathrm{vJS}}^{R}\left[p:q\right]:=\min_{c\in R}\left\{\frac{1}{2}D_{\mathrm{KL}}\left[p:c\right]+\frac{1}{2}D_{\mathrm{KL}}\left[q:c\right]\right\}.$$

In particular, Sibson [1] considered the normal information radius, i.e., the R-relative Jensen-Shannon divergence with $R=\{N\left(\mu ,\Sigma \right)\;:\;\left(\mu ,\Sigma \right)\in R^{d}\times P_{++}^{d}\}$, where $P_{++}^{d}$ denotes the open cone of d×d positive-definite matrices (positive-definite covariance matrices of Gaussian distributions). More generally, we may consider any exponential family E [2].

**Definition** **5** (Relative (R,M)-JS symmetrization of *D*)**.** *Let M be a mean and D a statistical distance. Subsequently, the relative (R,M)-JS symmetrization of D is:*

$$D_{M,R}^{\mathrm{vJS}}\left[p:q\right]:=\min_{c\in R}M\left(D[p:c],D[q:c]\right).$$

We obtain the relative Jensen-Shannon divergences when $D=D_{\mathrm{KL}}$.

**Example** **6.** *Grosse et al. [58] considered geometric and moment average paths for annealing. They proved that, when $p_{1}=p_{\theta_{1}}$ and $p_{2}=p_{\theta_{2}}$ belong to an exponential family [2]$E_{F}$ with cumulant function F, we have*

(98) $${\left(p_{1}p_{2}\right)}_{\beta }^{G}=\frac{p_{1}{\left(x\right)}^{1-\beta }p_{2}{\left(x\right)}^{\beta }}{\int p_{1}{\left(x\right)}^{1-\beta }p_{2}{\left(x\right)}^{\beta }d\mu \left(x\right)}=\arg\min_{c\in E_{F}}\left\{\left(1-\beta \right)D_{\mathrm{KL}}\left[c:p_{1}\right]+\beta D_{\mathrm{KL}}\left[c:p_{2}\right]\right\},$$

*and*

(99) $$p_{\bar{\eta }}=\arg\min_{c\in E_{F}}\left\{\left(1-\beta \right)D_{\mathrm{KL}}\left[p_{1}:c\right]+\beta D_{\mathrm{KL}}\left[c:p_{2}\right]\right\},$$

*where $\bar{\eta }=\left(1-\beta \right)\eta_{1}+\beta \eta_{2}$, $\eta_{i}=E_{p_{\theta_{i}}}\left[t\left(x\right)\right]$ (this is not an arithmetic mixture, but an exponential family density moment parameter that is a mixture of the parameters).*

The corresponding minima can be interpreted as relative skewed Jensen-Shannon symmetrization for the reverse KLD $D_{\mathrm{KL}}^{*}$ (Equation (98)) and the relative skewed Jensen-Shannon divergence (Equation (99)):

(100) $$\begin{array}{ccc} {D_{\mathrm{KL}}^{*}}_{A_{\beta },E_{F}}^{\mathrm{vJS}}\left[p_{1}:p_{2}\right] & = & \min_{c\in E_{F}}\left\{\left(1-\beta \right)D_{\mathrm{KL}}^{*}\left[p_{1}:c\right]+\beta D_{\mathrm{KL}}^{*}\left[p_{2}:c\right]\right\}, \end{array}$$

(101) $$\begin{array}{ccc} D_{A_{\beta },E_{F}}^{\mathrm{vJS}}\left[p_{1}:p_{2}\right] & = & \min_{c\in E_{F}}\left\{\left(1-\beta \right)D_{\mathrm{KL}}\left[c:p_{1}\right]+\beta D_{\mathrm{KL}}\left[c:p_{2}\right]\right\}, \end{array}$$

*where $A_{\beta }\left(a,b\right):=\left(1-\beta \right)a+\beta b$ is the weighted arithmetic mean for β∈(0,1).*

**Figure 1:**
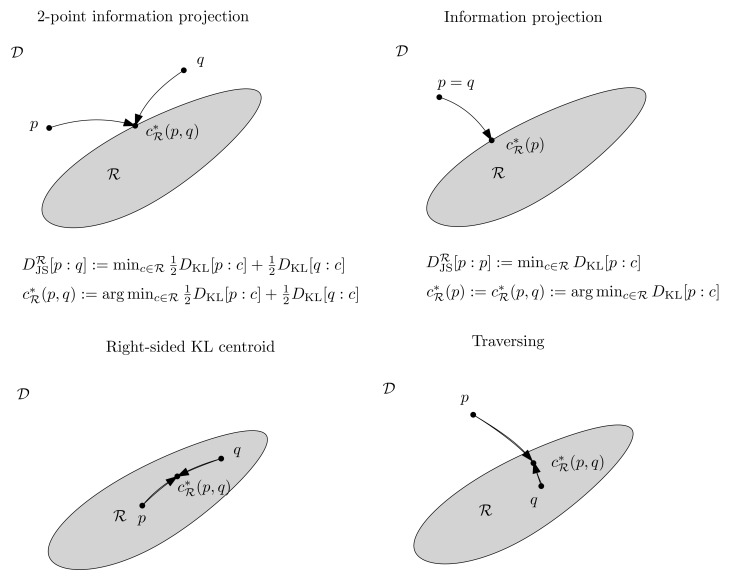
Illustrating several cases of the relative Jensen-Shannon divergence based on whether p∈R and q∈R or not.

Notice that, when p=q, we have $D_{M,R}^{\mathrm{vJS}}\left[p:p\right]=\min_{c\in R}D\left[p:c\right]$, which is the information projection [59] with respect to *D* of density *p* to the submanifold R. Thus, when p∉R, we have $D_{M,R}^{\mathrm{vJS}}\left[p:p\right]>0$, i.e., the relative JSDs are not proper divergences, since a proper divergence ensures that D[p:q]≥0 with equality if p=q. Figure 1 illustrates the main cases of the relative Jensen-Shannon divergenc between *p* and *q*: Either *p* and *q* are both inside or outside R, or one point is inside R, while the other point is outside R. When p=q, we get an information projection when both of the points are outside R, and $D_{\mathrm{vJS}}^{R}\left[p:p\right]=0$ when p∈R. When p,q∈R with p≠q, the value $D_{\mathrm{vJS}}^{R}\left[p:q\right]$ corresponds to the information radius (and the arg min to the right-sided Kullback–Leibler centroid).

### 4.2. Relative Jensen-Shannon Divergences: Applications to Density Clustering and Quantization

Let $D_{\mathrm{KL}}\left[p:q_{\theta }\right]$ be the Kullback–Leibler divergence between an *arbitrary* density *p* and a density $q_{\theta }$ of an exponential family $Q=\{q_{\theta }\;:\;\theta \in \Theta \}$. Let us canonically express [2,60] the density $q_{\theta }\left(x\right)$, as

$$q_{\theta }\left(x\right)=\exp\left(\theta^{⊤}t_{Q}\left(x\right)-F_{Q}\left(\theta \right)+k_{Q}\left(x\right)\right),$$

where $t_{Q}\left(x\right)$ denotes the sufficient statistics, $k_{Q}\left(x\right)$ is an auxiliary carrier measure term (e.g., k(x)=0 for the Gaussian family and k(x)=log(x) for the Rayleigh family [60]), and $F_{Q}\left(\theta \right)$ the cumulant function. Assume that we know in closed-form the following quantities:

- $m_{p}:=E_{p}\left[t_{Q}\left(x\right)\right]=\int p\left(x\right)t_{Q}\left(x\right)d\mu \left(x\right)$ and
- the Shannon entropy h[p]=−∫p(x)logp(x)dμ(x) of *p*.

Subsequently, we can express the KLD using a semi-closed-form formula.

**Proposition** **4.** *Let $q_{\theta }\in Q$ be a density of an exponential family and p an arbitrary density with $m_{p}=E_{p}\left[t_{Q}\left(x\right)\right]$. Subsequently, the Kullback–Leibler divergence between p and $q_{\theta }$ is expressed as:*

(102) $$D_{\mathrm{KL}}\left[p:q_{\theta }\right]=F_{Q}\left(\theta \right)-m_{p}^{⊤}\theta -E_{p}\left[k_{Q}\left(x\right)\right]-h\left[p\right],$$

*where $h\left[p:q_{\theta }\right]=F_{Q}\left(\theta \right)-m_{p}^{⊤}\theta -E_{p}\left[k_{Q}\left(x\right)\right]$ is the cross-entropy between p and $q_{\theta }$.*

**Proof.**  The proof is straightforward since $\log q_{\theta }\left(x\right)=\theta^{⊤}t_{Q}\left(x\right)-F_{Q}\left(\theta \right)+k_{Q}\left(x\right)$. Therefore, we have:

(103) $$\begin{array}{ccc} D_{\mathrm{KL}}\left[p:q_{\theta }\right] & = & h\left[p:q_{\theta }\right]-h\left[p\right], \end{array}$$

(104) $$\begin{array}{ccc} & = & -\int_{X}p\left(x\right)\log q_{\theta }\left(x\right)d\mu \left(x\right)-h\left[p\right], \end{array}$$

(105) $$\begin{array}{ccc} & = & F_{Q}\left(\theta \right)-m_{p}^{⊤}\theta -E_{p}\left[k_{Q}\left(x\right)\right]-h\left[p\right]. \end{array}$$

 □

**Example** **7.** *For example, when $q_{\theta }=q_{\mu ,\Sigma }$ is the density of a multivariate Gaussian distribution N(μ,Σ) (with $k_{N}\left(x\right)=0$), we have*

(106) $$D_{\mathrm{KL}}\left[p:q_{\mu ,\Sigma }\right]=\frac{1}{2}\left(\log|2\pi \Sigma |+{\left(\mu -m\right)}^{⊤}\Sigma^{-1}\left(\mu -m\right)+\mathrm{tr}\left(\Sigma^{-1}S\right)\right)-h\left[p\right],$$

*where $m=\mu \left(p\right)=E_{p}\left[X\right]$ and $S=\mathrm{Cov}\left(p\right):=E_{p}\left[XX^{⊤}\right]-E_{p}\left[X\right]E_{p}{\left[X\right]}^{⊤}$.*

The formula of Proposition 4 is said in semi-closed-form, because it relies on knowing both the entropy *h* of *p* and the sufficient statistic moments $E_{p}\left[t_{Q}\left(x\right)\right]$. Yet, this semi-closed formula may prove to be useful in practice: For example, we can answer the comparison predicate

“Is $D_{\mathrm{KL}}\left[p:q_{\theta_{1}}\right]\geq D_{\mathrm{KL}}\left[p:q_{\theta_{2}}\right]$ or not?”

by checking whether $F_{Q}\left(\theta_{1}\right)-F_{Q}\left(\theta_{2}\right)-m_{p}^{⊤}\left(\theta_{1}-\theta_{2}\right)\geq 0$ or not (i.e., the terms $-E_{p}\left[k_{Q}\left(x\right)\right]-h\left[p\right]$ in Equation (102) cancel out). Thus, we get a closed-form predicate, although $D_{\mathrm{KL}}$ is only known in semi-closed-form. This KLD comparison predicate shall be used later on when clustering densities with respect to centroids in Section 4.2.

**Remark** **1.** *Note that when Y=f(X) for an invertible and differentiable transformation f then we have $h\left[Y\right]=h\left[X\right]+E_{X}\left[\log\right|J_{f}\left(X\right)\left|\right]$ where $J_{f}$ denotes the Jacobian matrix. For example, when Y=f(X)=AX, we have h[Y]=h[X]+log|A|.*

When *p* belongs to an exponential family P (P may be different from Q) with cumulant function $F_{P}$, sufficient statistics $t_{P}\left(x\right)$, auxiliary carrier term $k_{P}\left(x\right)$, and natural parameter θ, we have the entropy [61] expressed, as follows:

(107) $$\begin{array}{ccc} h[p] & = & F_{P}\left(\theta \right)-\theta^{⊤}\nabla F_{P}\left(\theta \right)-E_{p}\left[k_{P}\left(x\right)\right], \end{array}$$

(108) $$\begin{array}{ccc} & = & -F_{P}^{*}\left(\eta \right)-E_{p}\left[k_{P}\left(x\right)\right], \end{array}$$

where $F_{P}^{*}\left(\eta \right)=\theta^{⊤}\nabla F\left(\theta \right)-F\left(\theta \right)$ is the Legendre transform of F(θ) and η=η(θ)=∇F(θ) is called the moment parameter since we have $\eta \left(\theta \right)=E_{p}\left[t_{P}\left(x\right)\right]$ [2,60].

It follows the following proposition refining Proposition 4 when $p=p_{\theta }\in P$:

**Proposition** **5.** *Let $p_{\theta }$ be a density of an exponential family P and $q_{\theta^{\prime }}$ be a density of an exponential family Q. Subsequently, the Kullback–Leibler divergence between $p_{\theta }$ and $q_{\theta^{\prime }}$ is expressed as:*

(109) $$D_{\mathrm{KL}}\left[p_{\theta }:q_{\theta^{\prime }}\right]=F_{Q}\left(\theta^{\prime }\right)+F_{P}^{*}\left(\eta \right)-E_{p_{\theta }}{\left[t_{Q}\left(x\right)\right]}^{⊤}\theta^{\prime }+E_{p_{\theta }}\left[k_{P}\left(x\right)-k_{Q}\left(x\right)\right].$$

**Proof.**  We have

(110) $$\begin{array}{ccc} D_{\mathrm{KL}}\left[p_{\theta }:q_{\theta^{\prime }}\right] & = & h\left[p_{\theta }:q_{\theta^{\prime }}\right]-h\left[p_{\theta }\right], \end{array}$$

(111) $$\begin{array}{ccc} & = & F_{Q}\left(\theta^{\prime }\right)-m_{p_{\theta }}^{⊤}\theta^{\prime }-E_{p_{\theta }}\left[k_{Q}\left(x\right)\right]+F_{P}^{*}\left(\eta \right)+E_{p_{\theta }}\left[k_{P}\left(x\right)\right], \end{array}$$

(112) $$\begin{array}{ccc} & = & F_{Q}\left(\theta^{\prime }\right)+F_{P}^{*}\left(\eta \right)-E_{p_{\theta }}{\left[t_{Q}\left(x\right)\right]}^{⊤}\theta^{\prime }+E_{p_{\theta }}\left[k_{P}\left(x\right)-k_{Q}\left(x\right)\right]. \end{array}$$

 □

In particular, when *p* and *q* belong both to the same exponential family (i.e., P=Q with $k_{P}\left(x\right)=k_{Q}\left(x\right)$), we have $F\left(\theta \right):=F_{P}\left(\theta \right):=F_{Q}\left(\theta \right)$ and $E_{p_{\theta }}\left[t_{Q}\left(x\right)\right]=\nabla F\left(\theta \right)=:\eta$, and

$$D_{\mathrm{KL}}\left[p_{\theta }:q_{\theta^{\prime }}\right]=F\left(\theta^{\prime }\right)+F^{*}\left(\eta \right)-\theta^{\prime ⊤}\eta .$$

This last equation is the Fenchel–Young divergence in Bregman manifolds [34,62] (called dually flat spaces in information geometry [10]). Thus the divergence can be rewritten as equivalent dual Bregman divergences:

(113) $$\begin{array}{ccc} D_{\mathrm{KL}}\left[p_{\theta }:q_{\theta^{\prime }}\right] & = & F\left(\theta^{\prime }\right)+F^{*}\left(\eta \right)-\eta^{⊤}\theta^{\prime }, \end{array}$$

(114) $$\begin{array}{ccc} & = & B_{F}\left(\theta^{\prime }:\theta \right), \end{array}$$

(115) $$\begin{array}{ccc} & = & B_{F^{*}}\left(\eta :\eta^{\prime }\right), \end{array}$$

where $\eta^{\prime }=\nabla F\left(\theta^{\prime }\right)$.

Notice that $D_{\mathrm{KL}}\left[p_{\theta }:Q\right]:=\min_{\theta^{\prime }\in \Theta^{\prime }}D_{\mathrm{KL}}\left[p_{\theta }:q_{\theta^{\prime }}\right]$ is unique and can be calculated as $\eta^{\prime }=\nabla F_{Q}\left(\theta^{\prime }\right)=E_{p_{\theta }}\left[t_{Q}\left(x\right)\right]$.

Let us report two examples of calculations of the KLD between two densities of two exponential families.

**Example** **8.** *For the first exponential family, consider the family of Laplacian distributions:*

$$P=L=\left\{p_{\sigma }\left(x\right):=\frac{1}{2\sigma }\exp\left(-\frac{\left|x\right|}{\sigma }\right)\;:\;\sigma >0\right\}.$$

The canonical decomposition of the density yields $t_{L}\left(x\right)=\left|x\right|$, $\theta =-\frac{1}{\sigma }$, $k_{L}\left(x\right)=0$, and $F_{L}\left(\theta \right)=\log\frac{2}{-\theta }$. (i.e., $F_{L}\left(\theta \left(\sigma \right)\right)=\log2\sigma$). It follows that $\eta \left(\theta \right)=F_{L}^{\prime }\left(\theta \right)=-\frac{1}{\theta }$ (η(σ)=σ=E[|x|]), $\theta \left(\eta \right)=-\frac{1}{\eta }$, and $F_{L}^{*}\left(\eta \right)=-1-\log\left(2\eta \right)$ and, therefore, $F_{L}^{*}\left(\eta \left(\sigma \right)\right)=-1-\log\left(2\sigma \right)$.

*For the second family, consider the exponential family of zero-centered Gaussian distributions:*

$$Q=N_{0}=\left\{q_{\sigma^{\prime }}\left(x\right)=\frac{1}{\sqrt{2\pi {\left(\sigma^{\prime }\right)}^{2}}}\exp\left(-\frac{x^{2}}{2{\left(\sigma^{\prime }\right)}^{2}}\right)\right\}.$$

*We have $t_{N_{0}}\left(x\right)=x^{2}$, $k_{N_{0}}\left(x\right)=0$, $\theta^{\prime }=-\frac{1}{2{\left(\sigma^{\prime }\right)}^{2}}$, and $F_{N_{0}}\left(\sigma^{\prime }\right)=\frac{1}{2}\log\left(2\pi {\left(\sigma^{\prime }\right)}^{2}\right)$.*

*Moreover, let us calculate $E_{p_{\sigma }}\left[t_{N_{0}}\left(x\right)\right]=E_{p_{\sigma }}\left[x^{2}\right]=2\sigma^{2}$. Subsequently, we can calculate the Kullback–Leibler divergence between $p_{\sigma }∼L\left(\sigma \right)$ and $q_{\sigma^{\prime }}∼N_{0}\left(\sigma^{\prime }\right)$, as follows:*

(116) $$\begin{array}{ccc} D_{\mathrm{KL}}\left[p_{\sigma }:q_{\sigma^{\prime }}\right] & = & F_{Q}\left(\theta^{\prime }\left(\sigma^{\prime }\right)\right)+F_{P}^{*}\left(\eta \left(\sigma \right)\right)-E_{p_{\sigma }}{\left[t_{Q}\left(x\right)\right]}^{⊤}\theta^{\prime }\left(\sigma^{\prime }\right)+E_{p_{\sigma }}\left[k_{P}\left(x\right)-k_{Q}\left(x\right)\right], \end{array}$$

(117) $$\begin{array}{ccc} & = & \frac{1}{2}\log\left(2\pi {\left(\sigma^{\prime }\right)}^{2}\right)-1-\log\left(2\sigma \right)-2\sigma^{2}\left(-\frac{1}{2{\left(\sigma^{\prime }\right)}^{2}}\right), \end{array}$$

(118) $$\begin{array}{ccc} & = & \log\left(\frac{\sigma^{\prime }}{\sigma }\right)+{\left(\frac{\sigma }{\sigma^{\prime }}\right)}^{2}+\frac{1}{2}\log\left(\frac{\pi }{2}\right)-1. \end{array}$$

*Notice that $D_{\mathrm{KL}}\left[p_{\sigma }:q_{\sigma^{\prime }}\right]\geq 0$, but never 0 since the P∩Q=∅.*

Let us now compute the reverse Kullback–Leibler divergence $D_{\mathrm{KL}}\left[q_{\sigma^{\prime }}:p_{\sigma }\right]$. We first calculate $E_{q_{\sigma^{\prime }}}\left[t_{L}\left(x\right)\right]=E_{q_{\sigma^{\prime }}\left(\sigma^{\prime }\right)}\left[|x|\right]=\sqrt{\frac{2}{\pi }}\sigma^{\prime }$. Since $F_{Q}\left(\theta^{\prime }\right)=\frac{1}{2}\log\left(\frac{\pi }{-\theta^{\prime }}\right)$, we have $\eta^{\prime }\left(\theta^{\prime }\right)=F_{Q}^{\prime }\left(\theta^{\prime }\right)=-\frac{1}{2\theta^{\prime }}$. Thus $\eta^{\prime }\left(\sigma^{\prime }\right)={\left(\sigma^{\prime }\right)}^{2}$ and $F_{Q}^{*}\left(\eta^{\prime }\right)=-\frac{1}{2}-\frac{1}{2}\log\left(2\pi \eta \right)$. Therefore, we get $F_{Q}^{*}\left(\eta^{\prime }\left(\sigma^{\prime }\right)\right)=-h\left[q_{\sigma^{\prime }}\right]=-\frac{1}{2}\log\left(2\pi e{\left(\sigma^{\prime }\right)}^{2}\right)$.

*It follows that*

(119) $$\begin{array}{ccc} D_{\mathrm{KL}}\left[q_{\sigma^{\prime }}:p_{\sigma }\right] & = & F_{P}\left(\theta \left(\sigma \right)\right)+F_{Q}^{*}\left(\eta^{\prime }\left(\sigma^{\prime }\right)\right)-E_{q_{\theta^{\prime }}}{\left[t_{P}\left(x\right)\right]}^{⊤}\theta \left(\sigma \right)+E_{q_{\theta^{\prime }}}\left[k_{P}\left(x\right)-k_{Q}\left(x\right)\right], \end{array}$$

(120) $$\begin{array}{ccc} & = & \log\left(2\sigma \right)-\frac{1}{2}\log\left(2\pi e{\left(\sigma^{\prime }\right)}^{2}\right)-\sqrt{\frac{2}{\pi }}\sigma^{\prime }\times \left(-\frac{1}{\sigma }\right), \end{array}$$

(121) $$\begin{array}{ccc} & = & \sqrt{\frac{2}{\pi }}\frac{\sigma^{\prime }}{\sigma }+\log\left(\frac{\sigma }{\sigma^{\prime }}\right)-\frac{1}{2}\log\left(\frac{\pi }{2}e\right). \end{array}$$

*Again, we have $D_{\mathrm{KL}}\left[q_{\sigma^{\prime }}:p_{\sigma }\right]\geq 0$, but never 0, because P∩Q=∅.*

**Example** **9.** *Let us use the formula of Equation (109) to calculate the KLD between two Weibull distributions [63]. A Weibull distribution of shape κ>0 and scale σ>0 has a density defined on X=[0,∞), as follows:*

$$p_{\kappa ,\sigma }^{\mathrm{Wei}}\left(x\right):=\frac{\kappa }{\sigma }{\left(\frac{x}{\sigma }\right)}^{\kappa -1}\exp\left(-{\left(\frac{x}{\sigma }\right)}^{\kappa }\right).$$

*For a fixed shape κ, the set of Weibull distributions $W_{\kappa }:=\left\{p_{\kappa ,\sigma }^{\mathrm{Wei}}\;:\;\sigma >0\right\}$ form an exponential family with natural parameter $\theta =-\frac{1}{\sigma^{\kappa }}$, sufficient statistic $t_{\kappa }\left(x\right)=x^{\kappa }$, auxiliary carrier term $k_{\kappa }\left(x\right)=\left(\kappa -1\right)\log x+\log\kappa$, and cumulant function $F_{\kappa }\left(\theta \right)=-\log\left(-\theta \right)$ (so that $F_{\kappa }\left(\theta \left(\sigma \right)\right)=F_{\kappa }\left(\sigma \right)=\kappa \log\sigma$):*

$$p_{\kappa ,\sigma }^{\mathrm{Wei}}\left(x\right):=\exp\left(-\frac{1}{\sigma^{\kappa }}x^{k}+\log\frac{1}{\sigma^{\kappa }}+k\left(x\right)\right).$$

*We recover the exponential family of exponential distributions of rate parameter $\lambda =\frac{1}{\sigma }$ when κ=1:*

$$\begin{array}{ccc} p_{\lambda }^{\mathrm{Exp}}\left(x\right) & = & p_{1,\sigma }^{\mathrm{Wei}}\left(x\right)=\frac{1}{\sigma }\exp\left(-\frac{x}{\sigma }\right),\\ & = & \lambda \exp\left(-\lambda x\right), \end{array}$$

*and the exponential family of Rayleigh distributions when κ=2 with scale parameter $\sigma_{\mathrm{Ray}}=\frac{\sigma }{\sqrt{2}}$:*

$$\begin{array}{ccc} p_{\sigma_{\mathrm{Ray}}}^{\mathrm{Ray}}\left(x\right) & = & p_{2,\sigma }^{\mathrm{Wei}}\left(x\right)=\frac{2x}{\sigma^{2}}\exp\left(-\frac{x^{2}}{\sigma^{2}}\right),\\ & = & \frac{x}{\sigma_{\mathrm{Ray}}^{2}}\exp\left(-\frac{x^{2}}{2\sigma_{\mathrm{Ray}}^{2}}\right). \end{array}$$

*Now, assume that we are given the differential entropy of the Weibull distributions [64] (pp. 155–156):*

$$h\left[p_{\kappa_{1},\sigma_{1}}^{\mathrm{Wei}}\right]=\gamma \left(1-\frac{1}{\kappa_{1}}\right)+\log\frac{\sigma_{1}}{\kappa_{1}}+1,$$

*where γ≈0.5772156649 is the Euler–Mascheroni constant, and the Weibull raw moments [64] (p. 155):*

$$m=E_{p_{\kappa_{1},\sigma }^{\mathrm{Wei}}}\left[x^{\kappa_{2}}\right]=\sigma_{1}^{\kappa_{2}}\Gamma \left(1+\frac{\kappa_{2}}{\kappa_{1}}\right),$$

where $\Gamma \left(x\right)=\int_{0}^{\infty }t^{x-1}e^{-t}dt$ is the gamma function (with Γ(n)=(n−1)! for integers n). Because $h\left[p_{\kappa ,\sigma }^{\mathrm{Wei}}\right]=F_{\kappa }\left(\theta \right)-\theta^{⊤}\nabla F_{\kappa }\left(\theta \right)-E_{p_{\kappa ,\sigma }^{\mathrm{Wei}}}\left[k_{\kappa }\left(x\right)\right]=-F_{\kappa }^{*}\left(\eta \right)-E_{p_{\kappa ,\sigma }^{\mathrm{Wei}}}\left[k_{\kappa }\left(x\right)\right]$, we deduce that

$$E_{p_{\kappa ,\sigma }^{\mathrm{Wei}}}\left[k_{\kappa }\left(x\right)\right]=-F_{\kappa }^{*}\left(\eta \right)-h\left[p_{\kappa ,\sigma }^{\mathrm{Wei}}\right],$$

where $F_{\kappa }^{*}\left(\eta \right)$ is the Legendre transform of $F_{\kappa }\left(\theta \right)$ and $\eta \left(\theta \right)=\nabla F_{\kappa }\left(\theta \right)=-\frac{1}{\theta }=E\left[t\left(x\right)\right]=E\left[x^{\kappa }\right]$. We have $\theta \left(\eta \right)=\nabla F_{\kappa }^{*}\left(\eta \right)=-\frac{1}{\eta }$ and $F_{\kappa }^{*}\left(\eta \right)=\eta^{⊤}\nabla F_{\kappa }^{*}\left(\eta \right)-F_{\kappa }\left(\nabla F_{\kappa }^{*}\left(\eta \right)\right)=-1-\log\eta$. It follows that

$$E_{p_{\kappa ,\sigma }^{\mathrm{Wei}}}\left[k_{\kappa }\left(x\right)\right]=1+\log\left(\sigma \Gamma \left(1+\frac{1}{\kappa }\right)\right)-\gamma \left(1-\frac{1}{\kappa }\right)-\log\frac{\sigma }{\kappa }+1.$$

*Therefore, we deduce that the logarithmic moment of $p_{\kappa_{1},\sigma }^{\mathrm{Wei}}$ is:*

$$E_{p_{\kappa_{1},\sigma }^{\mathrm{Wei}}}\left[\log x\right]=-\frac{\gamma }{\kappa_{1}}+\log\sigma_{1}.$$

*This coincides with the explicit definite integral calculation reported in [63].*

*Subsequently, we calculate the KLD between two Weibull distributions using Equation (109), as follows:*

(122) $$\begin{array}{ccc} D_{\mathrm{KL}}\left[p_{\kappa_{1},\sigma_{1}}^{\mathrm{Wei}}:p_{\kappa_{2},\sigma_{2}}^{\mathrm{Wei}}\right] & = & F_{\kappa_{2}}\left(\theta^{\prime }\right)+F_{\kappa_{1}}^{*}\left(\eta \right)-E_{p_{\kappa_{1},\sigma_{1}}}{\left[x^{\kappa_{2}}\right]}^{⊤}\theta^{\prime }+E_{p_{\kappa_{1},\sigma_{1}}}\left[k_{\kappa_{1}}\left(x\right)-k_{\kappa_{2}}\left(x\right)\right] \end{array}$$

(123) $$\begin{array}{ccc} & = & \log\frac{\kappa_{1}}{\sigma_{1}^{\kappa_{1}}}-\log\frac{\kappa_{2}}{\sigma_{2}^{\kappa_{2}}}+\left(\kappa_{1}-\kappa_{2}\right)\left[\log\sigma_{1}-\frac{\gamma }{\kappa_{1}}\right]+{\left(\frac{\sigma_{1}}{\sigma_{2}}\right)}^{\kappa_{2}}\Gamma \left(\frac{\kappa_{2}}{\kappa_{1}}+1\right)-1\\ \; \end{array}$$

*since we have the following terms:*

$$\begin{array}{ccc} F_{\kappa_{2}}\left(\theta^{\prime }\right) & = & \log\sigma_{2}^{\kappa_{2}},\\ F_{\kappa_{1}}^{*}\left(\eta \right) & = & -1-\log\sigma_{1}^{\kappa_{1}},\\ -E_{p_{\kappa_{1},\sigma_{1}}}{\left[x^{\kappa_{2}}\right]}^{⊤}\theta^{\prime } & = & \frac{1}{\sigma_{2}^{\kappa_{2}}}\sigma_{1}^{\kappa_{2}}\Gamma \left(1+\frac{\kappa_{2}}{\kappa_{1}}\right)\\ E_{p_{\kappa_{1},\sigma_{1}}}\left[k_{\kappa_{1}}\left(x\right)-k_{\kappa_{2}}\left(x\right)\right] & = & \left(\kappa_{1}-\kappa_{2}\right)E_{p_{\kappa_{1},\sigma_{1}}}\left[\log x\right]+\log\frac{\kappa_{1}}{\kappa_{2}},\\ & = & \log\frac{\kappa_{1}}{\kappa_{2}}+\left(\kappa_{1}-\kappa_{2}\right)\left(\log\sigma_{1}-\frac{\gamma }{\kappa_{1}}\right). \end{array}$$

*This formula matches the formula reported in [63].*

*When $\kappa_{1}=\kappa_{2}=1$, we recover the ordinary KLD formula between two exponential distributions [60] with $\lambda_{i}=\frac{1}{\sigma_{i}}$ since Γ(2)=(2−1)!=1:*

(124) $$\begin{array}{ccc} D_{\mathrm{KL}}\left[p_{1,\sigma_{1}}^{\mathrm{Wei}}:p_{1,\sigma_{2}}^{\mathrm{Wei}}\right] & = & \log\frac{\sigma_{2}}{\sigma_{1}}+\frac{\sigma_{1}}{\sigma_{2}}-1, \end{array}$$

(125) $$\begin{array}{ccc} & = & \frac{\lambda_{2}}{\lambda_{1}}-\log\frac{\lambda_{2}}{\lambda_{1}}-1. \end{array}$$

*When $\kappa_{1}=\kappa_{2}=2$, we recover the ordinary KLD formula between two Rayleigh distributions [60], with $\sigma_{\mathrm{Ray}}=\frac{\sigma }{\sqrt{2}}$:*

(126) $$\begin{array}{ccc} D_{\mathrm{KL}}\left[p_{2,\sigma_{1}}^{\mathrm{Wei}}:p_{2,\sigma_{2}}^{\mathrm{Wei}}\right] & = & \log\left(\frac{\sigma_{2}^{2}}{\sigma_{1}^{2}}\right)+\frac{\sigma_{1}^{2}}{\sigma_{2}^{2}}-1, \end{array}$$

(127) $$\begin{array}{ccc} & = & \log\left(\frac{{\sigma_{\mathrm{Ray}}}_{2}^{2}}{{\sigma_{\mathrm{Ray}}}_{1}^{2}}\right)+\frac{{\sigma_{\mathrm{Ray}}}_{1}^{2}}{{\sigma_{\mathrm{Ray}}}_{2}^{2}}-1. \end{array}$$

*The formulae of Equations (127) and (126) are linked by the fact that if X∼Exp(λ) and $Y=\sqrt{X}$ then $Y∼\mathrm{Ray}\left(\frac{1}{\sqrt{2\lambda }}\right)$, and f-divergences [65], including the Kullback–Leibler divergence are invariant by a differentiable transformation [66].*

Jeffreys’ divergence symmetrizes the KLD divergence, as follows:

(128) $$D_{J}\left[p:q\right]:=D_{\mathrm{KL}}\left[p:q\right]+D_{\mathrm{KL}}\left[q:p\right]=2A\left(D_{\mathrm{KL}}\left[p:q\right],D_{\mathrm{KL}}\left[q:p\right]\right).$$

The Jeffreys divergence between two densities of different exponential families P and Q is

(129) $$D_{J}\left[p_{\theta }:q_{\theta^{\prime }}\right]=\theta^{\prime ⊤}\left(\eta^{\prime }-E_{p_{\theta }}\left[t_{Q}\left(x\right)\right]\right)+\theta^{⊤}\left(\eta -E_{q_{\theta^{\prime }}}\left[t_{P}\left(x\right)\right]\right)+E_{p_{\theta }}\left[k_{P}\left(x\right)-k_{Q}\left(x\right)\right]+E_{q_{\theta^{\prime }}}\left[k_{Q}\left(x\right)-k_{P}\left(x\right)\right].$$

When P=Q, we have $E_{p_{\theta }}\left[t_{Q}\left(x\right)\right]=\eta$ and $E_{q_{\theta^{\prime }}}\left[t_{P}\left(x\right)\right])=\eta^{\prime }$, so that we find the usual expression of the Jeffreys divergence between two densities of an exponential family:

(130) $$D_{J}\left[p_{\theta }:p_{\theta^{\prime }}\right]={\left(\theta^{\prime }-\theta \right)}^{⊤}\left(\eta^{\prime }-\eta \right).$$

To find the best density $q_{\theta }$ approximating *p* by minimizing $\min_{\theta }D_{\mathrm{KL}}\left[p:q_{\theta }\right]$, we solve ∇F(θ)=η=m and, therefore, $\theta =\nabla F^{*}\left(m\right)={\left(\nabla F\right)}^{-1}\left(m\right)$, where $F^{*}\left(\eta \right)=E_{q_{\eta }}\left[\log q_{\eta }\left(m\right)\right]$, with $F^{*}$ denoting the Legendre–Fenchel convex conjugate [2]. In particular, when $p=\sum w_{i}p_{\theta_{i}}$ is a mixture of EFs (with $m=E_{p}\left[t\left(x\right)\right]=\sum w_{i}\eta_{i}$ with $\eta_{i}=E_{p_{\theta_{i}}}\left[t\left(x\right)\right]$ thanks to the linearity of the expectation), then the best density of the EF simplifying *p* is

(131) $$\begin{array}{ccc} \min_{\theta }D_{\mathrm{KL}}\left[p:q_{\theta }\right] & = & \min_{\theta }F\left(\theta \right)-m^{⊤}\theta , \end{array}$$

(132) $$\begin{array}{ccc} & = & \min_{\theta }F\left(\theta \right)-\sum w_{i}\eta_{i}^{⊤}\theta . \end{array}$$

Taking the gradient with respect to θ, we have $\nabla F\left(\theta \right)=\eta =\sum w_{i}\eta_{i}$. This yields another proof without the Pythagoras theorem [67,68].

**Proposition** **6.** Let $m\left(x\right)=\sum w_{i}p_{\theta_{i}}\left(x\right)$ be a mixture with components that belong to an exponential family with cumulant function F. Subsequently, $\theta^{*}=\arg_{\theta }\min_{\theta }D_{\mathrm{KL}}\left[p:q_{\theta }\right]$ is $\nabla F^{*}\left(\sum_{i=1}^{n}w_{i}\eta_{i}\right)$, where the $\eta_{i}=\nabla F\left(\theta_{i}\right)$ are the moment parameters of the mixture components.

Consider the following two problems:

**Problem** **1** (Density clustering)**.** *Given a set of n weighted densities $\left(w_{1},p_{1}\right),\ldots ,\left(w_{n},p_{n}\right)$, partition them into k clusters $C_{1},\ldots ,C_{k}$ in order to minimize the k-centroid objective function with respect to a statistical divergence D: $\sum_{i=1}^{n}w_{i}\min_{l\in \{1,\ldots ,k\}}D\left[p_{i}:c_{l}\right]$, where $c_{l}$ denotes the centroid of cluster $C_{l}$ for l∈{1,…,k}.*

For example, when all the densities $p_{i}$’s are isotropic Gaussians, we recover the *k*-means objective function [69].

**Problem** **2** (Mixture component quantization)**.** *Given a statistical mixture $m\left(x\right)=\sum_{i=1}^{n}w_{i}$$p_{i}\left(x\right)$, quantize the mixture components into k densities $q_{1},\ldots ,q_{k}$ in order to minimize $\sum_{i}w_{i}$$\min_{l\in \{1,\ldots ,k\}}D\left[p_{i}:q_{l}\right]$.*

Notice that, in Problem 1, the input densities $p_{i}$’s may be mixtures, i.e., $p_{i}\left(x\right)=\sum_{j=1}^{n_{i}}w_{i,j}p_{i,j}\left(x\right)$. Using the relative information radius, we can cluster a set of distributions (potentially mixtures) into an exponential family mixture, or quantize an exponential family mixture. Indeed, we can implement an extension of *k*-means [69] with *k*-centers $q_{\theta_{i}}$, to assign density $p_{i}$ to cluster $C_{j}$ (with center $q_{j}$), we need to perform basic comparison tests $D_{\mathrm{KL}}\left[p_{i}:q_{\theta_{l}}\right]\geq D_{\mathrm{KL}}\left[p_{i}:q_{\theta_{j}}\right]$. Provided that the cumulant *F* of the exponential family is in closed-form, we do not need formula for the entropies $h(p_{i})$.

Clustering and quantization of densities/mixtures have been widely studied in the literature, see, for example, [70,71,72,73,74,75,76].

## 5. Conclusions

To summarize, the ordinary Jensen-Shannon divergence has been defined in three equivalent ways in the literature:

(133) $$\begin{array}{ccc} D_{\mathrm{JS}}\left[p,q\right] & := & \min_{c\in D}\frac{1}{2}\left(D_{\mathrm{KL}}\left[p:c\right]+D_{\mathrm{KL}}\left[q:c\right]\right), \end{array}$$

(134) $$\begin{array}{ccc} & = & \frac{1}{2}\left(D_{\mathrm{KL}}\left[p:\frac{p+q}{2}\right]+D_{\mathrm{KL}}\left[q:\frac{p+q}{2}\right]\right), \end{array}$$

(135) $$\begin{array}{ccc} & = & h\left[\frac{p+q}{2}\right]-\frac{h[p]+h[q]}{2}. \end{array}$$

The JSD Equation (133) was studied by Sibson in 1969 within the wider scope of information radius [1]: Sibson relied on the Rényi α-divergences (relative Rényi α-entropies [77]) and recovered the ordinary Jensen-Shannon divergence as a particular case of the α-information radius when α=1 and n=2 points. The α-information radii are related to generalized Bhattacharyya distances with respect to power means and the total variation distance in the limit case of α=∞.

Lin [4] investigated the JSD Equation (134) in 1991 with its connection to the JSD defined in Equation (134)). In Lin [4], the JSD is interpreted as the arithmetic symmetrization of the *K*-divergence [24]. Generalizations of the JSD based on Equation (134) were proposed in [23] using a generic mean instead of the arithmetic mean. One motivation was to obtain a closed-form formula for the geometric JSD between multivariate Gaussian distributions, which relies on the geometric mixture (see [30] for a use case of that formula in deep learning). Indeed, the ordinary JSD between Gaussians is not available in closed-form (not analytic). However, the JSD between Cauchy distributions admit a closed-form formula [78], despite the calculation of a definite integral of a log-sum term. Instead of using an abstract mean to define a mid-distribution of two densities, one may also consider the mid-point of a geodesic linking these two densities (the arithmetic means $\frac{p+q}{2}$ is interpreted as a geodesic midpoint). Recently, Li [79] investigated the transport Jensen-Shannon divergence as a symmetrization of the Kullback–Leibler divergence in the $L^{2}$-Wasserstein space. See Section 5.4 of [79] and the closed-form formula of Equation (18) obtained for the transport Jensen-Shannon divergence between two multivariate Gaussian distributions.

The generalization of the identity between the JSD of Equation (134) and the JSD of Equation (135) was studied while using a skewing vector in [18]. Although the JSD is a *f*-divergence [8,18], the Sibson-*M* Jensen-Shannon symmetrization of a distance does not belong, in general, to the class of *f*-divergences. The variational JSD definition of Equation (133) is implicit, while the definitions of Equations (134) and (135) are explicit because the unique optimal centroid $c^{*}=\frac{p+q}{2}$ has been plugged into the objective function that was minimized by Equation (133).

**Figure 2:**
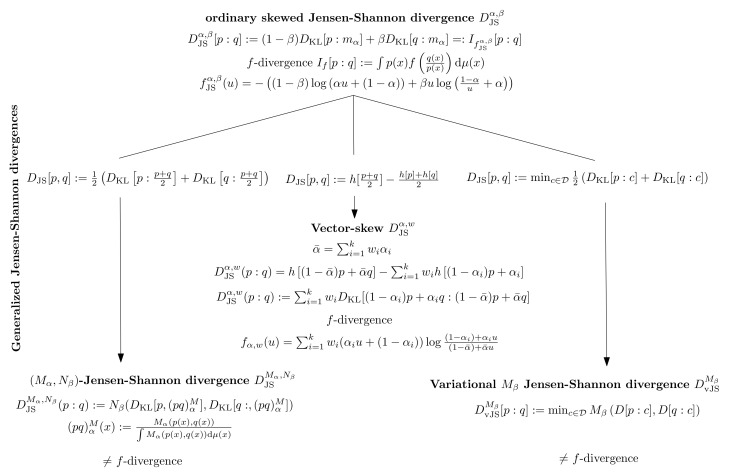
Three equivalent expressions of the ordinary (skewed) Jensen-Shannon divergence which yield three different generalizations.

In this paper, we proposed a generalization of the Jensen-Shannon divergence based on the variational definition of the ordinary Jensen-Shannon divergence based on the variational JSD definition of Equation (133): $D_{\mathrm{vJS}}\left[p:q\right]=\min_{c}\frac{1}{2}\left(D_{\mathrm{KL}}\left[p:c\right]+D_{\mathrm{KL}}\left[q:c\right]\right)$. We introduced the Jensen-Shannon symmetrization of an arbitrary divergence *D* by considering a generalization of the information radius with respect to an abstract weighted mean $M_{\beta }$: $D_{M}^{\mathrm{vJS}}\left[p:q\right]:=\min_{c}M_{\beta }\left(D\left[p:c\right],D\left[q:c\right]\right)$. Notice that, in the variational JSD, the mean $M_{\beta }$ is used for averaging divergence values, while the mean $M_{\alpha }$ in the $\left(M_{\alpha },N_{\beta }\right)$ JSD is used to define generic statistical mixtures. We also consider relative variational JS symmetrization when the centroid has to belong to a prescribed family of densities. For the case of exponential family, we showed how to compute the relative centroid in closed form, thus extending the pioneering work of Sibson, who considered the relative normal centroid used to calculate the relative normal information radius. Figure 2 illustrates the three generalizations of the ordinary skewed Jensen-Shannon divergence. Notice that, in general, the (M,N)-JSDs and the variational JDSs are not *f*-divergences (except in the ordinary case).

In a similar vein, Chen et al. [80] considered the following minimax symmetrization of the scalar Bregman divergence [81]:

(136) $$\begin{array}{ccc} B_{f}^{\mathrm{minmax}}\left(p,q\right) & := & \min_{c}\max_{\lambda \in [0,1]}\lambda B_{f}\left(p:c\right)+\left(1-\lambda \right)B_{f}\left(q:c\right), \end{array}$$

(137) $$\begin{array}{ccc} & = & \max_{\lambda \in [0,1]}\lambda B_{f}\left(p:\lambda p+\left(1-\lambda \right)q\right)+\left(1-\lambda \right)B_{f}\left(q:\lambda p+\left(1-\lambda \right)\right), \end{array}$$

(138) $$\begin{array}{ccc} & = & \lambda f(p)+(1-\lambda )f(q)-f(\lambda p+(1-\lambda )) \end{array}$$

where $B_{f}$ denotes the scalar Bregman divergence induced by a strictly convex and smooth function *f*:

(139) $$B_{f}\left(p:q\right)=f\left(p\right)-f\left(q\right)-\left(p-q\right)f^{\prime }\left(q\right).$$

They proved that $\sqrt{B_{f}^{\mathrm{minmax}}\left(p,q\right)}$ yields a metric when $3{\left(\log f^{\prime \prime }\right)}^{\prime \prime }\geq {\left({\left(\log f^{\prime \prime }\right)}^{\prime }\right)}^{2}$, and extend the definition to the vector case and conjecture that the square-root metrization still holds in the multivariate case. In a sense, this definition geometrically highlights the notion of radius, since the minmax optimization amount to find a smallest enclosing ball enclosing [82] the source distributions. The circumcenter, also called the Chebyshev center [83], is then the mid-distribution instead of the centroid for the information radius. The term "information radius” is well-suited to measure the distance between two points for an arbitrary distance *D*. Indeed, the JS-symmetrization of *D* is defined by $D^{\mathrm{JS}}\left[p:q\right]:=\min_{c}\left\{\frac{1}{2}D\left[p:c\right]+\frac{1}{2}D\left[q:c\right]\right\}$. When $D\left[p:q\right]=D_{E}\left[p:q\right]=∥p-q∥$ is the Euclidean distance, we have $c=\frac{p+q}{2}$, and $D\left[p:c\right]=D\left[q:c\right]=\frac{1}{2}∥p-q∥=:r$ (i.e., the radius being half of the diameter ∥p−q∥). Thus, $D_{E}^{\mathrm{JS}}\left[p:q\right]=r$; hence, the term chosen by Sibson [1] for $D^{\mathrm{JS}}$: information radius. Besides providing another viewpoint, variational definitions of divergences have proven to be useful in practice (e.g., for estimation). For example, a variational definition of the Rényi divergence generalizing the Donsker–Varadhan variational formula of the KLD is given in [84], which is used to estimate the Rényi Divergences.

## Data Availability

Data sharing not applicable.
